# New Neurons in the Post-ischemic and Injured Brain: Migrating or Resident?

**DOI:** 10.3389/fnins.2019.00588

**Published:** 2019-06-18

**Authors:** Nikolai M. Nemirovich-Danchenko, Marina Yu. Khodanovich

**Affiliations:** Laboratory of Neurobiology, Research Institute of Biology and Biophysics, Tomsk State University, Tomsk, Russia

**Keywords:** neurogenesis, neural stem cells, migration, stroke, traumatic brain injury, subventricular zone, striatum, cortex

## Abstract

The endogenous potential of adult neurogenesis is of particular interest for the development of new strategies for recovery after stroke and traumatic brain injury. These pathological conditions affect endogenous neurogenesis in two aspects. On the one hand, injury usually initiates the migration of neuronal precursors (NPCs) to the lesion area from the already existing, in physiological conditions, neurogenic niche – the ventricular-subventricular zone (V-SVZ) near the lateral ventricles. On the other hand, recent studies have convincingly demonstrated the local generation of new neurons near lesion areas in different brain locations. The striatum, cortex, and hippocampal CA1 region are considered to be locations of such new neurogenic zones in the damaged brain. This review focuses on the relative contribution of two types of NPCs of different origin, resident population in new neurogenic zones and cells migrating from the lateral ventricles, to post-stroke or post-traumatic enhancement of neurogenesis. The migratory pathways of NPCs have also been considered. In addition, the review highlights the advantages and limitations of different methodological approaches to the definition of NPC location and tracking of new neurons. In general, we suggest that despite the considerable number of studies, we still lack a comprehensive understanding of neurogenesis in the damaged brain. We believe that the advancement of methods for *in vivo* visualization and longitudinal observation of neurogenesis in the brain could fundamentally change the current situation in this field.

## Introduction

At present, it is well known that the production of new neurons in the mammalian brain is not restricted to the embryonic and early postnatal development stages, but occurs throughout the lifespan of animals. However, not all brain regions are equally capable of generating new neurons. There are two main neurogenic niches where neurogenesis persists: the ventricular-subventricular zone (V-SVZ) and the subgranular zone of the hippocampus (SGZ). By the term V-SVZ we mean a complex of brain regions that includes: the subventricular zone (SVZ) – the area stretching along the lateral wall of the lateral ventricles, the ventral extension of the lateral ventricles, and the posterior periventricular region (PPV). Several researchers have also considered these areas as a single neurogenic zone (Lim and Alvarez-Buylla, 2016; Mizrak et al., 2019). During adulthood, neurogenesis also occurs in several other brain regions, including the striatum (Luzzati et al., 2006, 2014) and cortex (Gould et al., 2002; Dayer et al., 2005). At the same time, in mice, the most well-studied mammalian species, under physiological conditions no neurogenesis occurs in the cortex (Ehninger and Kempermann, 2003) and striatum (Teramoto et al., 2003; Luzzati et al., 2011; Nato et al., 2015) or, probably, it occurs but only at a very low rate.

Some types of brain damage, such as ischemia or traumatic brain injury (TBI), stimulate neurogenesis in the brain (Sun, 2016; Marques et al., 2019). On the one hand, we know that the proliferation of neuronal precursors (NPCs) in the V-SVZ (the most active proliferative region of the brain) increases, and that these precursors migrate from their place of origin to the injury site (Nakatomi et al., 2002; Jin et al., 2003; Yamashita et al., 2006). On the other hand, many studies have shown that when the brain is damaged, new neurons are generated from local neural stem cells (NSCs) at the injury site (Ohira et al., 2010; Magnusson et al., 2014; Nato et al., 2015). This raises the question: do the NPCs that migrate from the V-SVZ play a major role in the injury-related neurogenesis, or is the role of resident NPCs more important? The answer to this question is of huge clinical significance, because it will determine treatment methods targeted either at the increase in localized neurogenesis near the injury site or at the increase in neurogenesis in – and migration from – the V-SVZ. Consequently, numerous studies have been carried out to establish the dynamics of proliferation and migration of NPCs in the brain in various conditions. In the present review we consider the studies with a focus on the features of neurogenesis in conditions of ischemia and TBI.

## Methods for Studying the Production and Migration of NPCs

Here we consider approaches to studying neurogenesis, paying particular attention to how the origin of new neurons can be established. In other words, whether new neurons have originated in the same area where they localize, or if they/their precursors have migrated there from another area. Methods for defining migration pathways of NPCs are also reviewed here.

### Research Into Spatial Distribution of Neuronal Precursors in Fixed Brain Tissues

Historically, the first studies that established the production of new neurons in the mammalian brain were performed using autoradiographic investigation of 3H-thymidine-labeled dividing cells, in particular, newborn neurons (Altman, 1962; Kaplan and Hinds, 1977; Goldman and Nottebohm, 2006). However, neurogenesis in the adult mammalian brain has not been acknowledged by the scientific community for a long time. The existence of neurogenesis was recognized only after methodological improvement, which promoted further studies. This improvement was replacement of radioactive analog of thymidine with bromodeoxiuridine (BrdU), which can be detected by immunohistochemistry (Abrous et al., 2005).

This approach, based on the administration of such proliferation markers as BrdU and other analogs of thymidine, still remains among the most widely used methods. BrdU can be administered intraperitoneally or with drinking water. It incorporates into the cells that are in S-phase at the time of administration. The timing of BrdU administration and subsequent killing of animals affect the interpretation of results. When BrdU is given systematically for several days or weeks, labeled cells born at different time points cannot be distinguished. Alternatively, a single dose (pulse) of BrdU labels the cells that are born at the time of the pulse. Then animals can be sacrificed either shortly after administration, or at a delayed time point (pulsed and pulse-chase paradigm, respectively). This approach allows for the tracing of a short-term or a long-term fate of a particular group of cells that are born at a certain time point (Dayer et al., 2005; Sundholm-Peters et al., 2005; Shapiro et al., 2009; Lugert et al., 2012).

In addition to BrdU, other nucleotide analogs can be used: -5-chloro-20-deoxyuridine (CldU), 5-iodo-20-deoxyuridine (IdU), and 5-ethynyl-20-deoxyuridine (EdU). The use of combination of several thymidine analogs within the same experiment gives additional possibilities. Labeling of dividing cells with two (Encinas et al., 2011) or even three (Podgorny et al., 2018) nucleotide analogs allows for measuring cell-division kinetics, and identifying and tracing subclasses of NSCs or NPCs. Assessment of the fraction of CldU/IdU double-labeled NPCs enabled Encinas et al. (2011) to determine the number of divisions, the length of S-phase, and the length of the division cycle of amplifying and quiescent neural progenitors in the dentate gyrus (DG).

The immunodetection of BrdU or other thymidine analogs in fixed brain slices, in combination with labeling the specific markers of immature (doublecortin or DCX) and mature (NeuN, MAP2, NSE) neurons, as well as NSCs (Nestin, SOX2), allows for indirect tracking of NPCs. Additionally, Ki67, a marker of cell divisions, is used to confirm the proliferation status and to identify the location of non-migrating progenitors (Abrous et al., 2005; Kuhn et al., 2016).

Although this approach facilitated the estimation of the proliferation and differentiation of NPCs, it provided only indirect evidence of neuroblast migration. The signs of migration can be: a distribution pattern of migrating neuroblasts (markers – DCX and sometimes PSA-NCAM) from the place of origin toward the place of destination (Palma-Tortosa et al., 2017), a density gradient of neuroblasts with high density at the presumed place of origin (Thored et al., 2006; Fukuzaki et al., 2015), as well as the change in this gradient at subsequent time points (Yan et al., 2007; Oya et al., 2009; Li et al., 2011). Also, in their works researchers pay due attention to the orientation of migrating cells. Neuroblasts stretch out their leading processes in the direction of the zone to which they are moving (Parent et al., 2002; Zhang et al., 2004; Dayer et al., 2005; Osman et al., 2011; Nakaguchi et al., 2012; Moraga et al., 2014). Moreover, migrating neuroblasts often integrate into chains, and the orientation of the chain also corresponds to the direction of migration (Parent et al., 2002; Zhang et al., 2004; Wang et al., 2007; Kuge et al., 2009; Yan et al., 2009b; Kazanis et al., 2013; Wan et al., 2016; Song et al., 2017). Alternatively, clustering cells with round cell bodies, lacking long leading processes and co-expressing DCX with proliferation marker Ki67 are considered as non-migrating local NPCs (Magnusson et al., 2014; Nato et al., 2015).

Studies of postmortem tissues are of great importance in research on human neurogenesis because this is the only way it can be investigated. BrdU labeling in combination with other neuronal markers enabled scientists to prove for the first time the existence of neurogenesis in humans (Eriksson et al., 1998). A more sophisticated technique of ^14^C-dating was proposed by Jonas Frisen’s group (Bhardwaj et al., 2006; Spalding et al., 2013). The technique is based on the fact that the ^14^C levels in the atmosphere have been decreasing at a known rate since a sharp rise in the ^14^C content caused by the extensive testing of nuclear weapons in the middle of the 20th centuary. Thus, comparison of the ^14^C-content in DNA with that in the atmosphere enabled the assessment of the age of cells. Isolation of cell nuclei from brain tissue with subsequent labeling with NeuN and the ^14^C content measuring by accelerator mass spectrometry allowed for evaluation of the turnover rate of neurons in the human hippocampus, cortex, olfactory bulb (OB), and striatum (Bhardwaj et al., 2006; Spalding et al., 2013; Ernst et al., 2014).

The above-mentioned studies have investigated neuroblasts distribution in brain slices. The limitation of this approach is a lack of information about whole brain tissues. In recent years, completely new techniques for *ex vivo* studies which potentially expand possibilities for the investigation of new neuron generation have been devised.

A promising technique called CLARITY (Chung and Deisseroth, 2013) was developed by the Deisseroth lab. This technique makes the entire brain transparent for any optical imaging via special chemical transformations of intact brain tissue. These transformations aim to remove the lipid component of the brain while retaining the protein and nucleic components in their native state. After transformation, the preserved components can either be immunohistochemically stained or initially fluorescent-labeled by genetic modification, thereby facilitating the performance of precise whole-brain imaging without dividing into slices. Imaging of the whole brain rather than of just separate brain slices could provide an entire picture of the spatial distribution and orientation of migrating NPCs.

Despite the unique possibility to obtain 3D images of brain structure at the molecular level, we did not find any research works that have used this method since it was published. The major limitations of this method are the complexity and timescales involved in the brain processing and immunostaining, as well as the toxicity of the reagents used (Jensen and Berg, 2017). Significant efforts have been made to improve this technique (Jensen and Berg, 2017) in order that it might be more extensively used in studies – including NPC tracking.

Another powerful technique, single-cell transcriptomics, is based on measuring gene expression at the level of individual cells in certain cell populations. It helps clarify the mechanisms of cell reprogramming into NSCs in non-neurogenic zones after injury and promotes a better understanding of the balance of NSC activation and quiescent state in the neurogenic niches (Llorens-Bobadilla et al., 2015). Cell subgroups identified by this technique can be compared to the known cell types using previously established marker genes; however, novel cell subtypes can also be discovered using single-cell data (Liu and Trapnell, 2016). Unlike immunofluorescent detection with antibodies, which is usually limited in the number of markers, single-cell transcriptomics allows for the simultaneous investigation of hundreds, or even thousands, of genes. The main limitation of this technique in studies on cell migration is the loss of the original spatial context. Currently, efforts are being made to overcome this limitation by using computational methods of 3D reconstruction (Satija et al., 2015). Combining single-cell transcriptomics with other single-cell techniques, such as fluorescent RNA FISH, provides an orthogonal method of quantifying transcript levels, and is often used to independently validate results from scRNA-seq data (Liu and Trapnell, 2016).

In general, neuroblast distribution, orientation, morphology and clustering are only indirect signs of migration. Additionally, this approach is limited only to estimation of the origin and migration direction of cells, which at a certain time point are migrating neuroblasts. However, we lack information on the origin of new cells that have already become mature neurons. In the next section, we will discuss methods that allow us to specifically trace the fate of cells of a particular origin, i.e., the cells that originated from the SVZ or cortex.

### Determining the Place/Time of NPCs Production by Chemical and Genetic Labeling

Local labeling of the cells within a particular region of the brain is a powerful method for tracing the migration of NPCs that are produced there. After labeling, the fate of the NPCs and their offspring can be examined at different time points. For example, these labeled cells can be found in a different brain region some time later, or they can stay at their place of origin. To determine a phenotype of stained cells, immunohistochemical detection of such cell type-specific proteins as DCX, calretinin, NeuN, MAP2, NSE, and Nestin is carried out (Nakatomi et al., 2002; Jin et al., 2003; Zhao et al., 2003; Goings et al., 2004; Ramaswamy et al., 2005; Zhang et al., 2005, 2011; Ohab et al., 2006; Yamashita et al., 2006; Kolb et al., 2007; Yang et al., 2007, 2008; Faiz et al., 2008, 2015; Hou et al., 2008; Lai et al., 2008; Liu et al., 2009; Kreuzberg et al., 2010; Li B. et al., 2010; Li L. et al., 2010; Ohira et al., 2010; Shimada et al., 2010; Yoshikawa et al., 2010; Bi et al., 2011; Grade et al., 2013; Saha et al., 2013; Magnusson et al., 2014; Duan et al., 2015).

Local injection of various tracers into the brain is widely used. In this method, the cells residing in the zone of injection, absorb the tracer; this then allows for tracking the fate of these cells, or their offspring at different post-injection time points. Tracers can be either a stain (Nakatomi et al., 2002; Jin et al., 2003; Zhao et al., 2003; Ramaswamy et al., 2005; Zhang et al., 2005; Faiz et al., 2008; Hou et al., 2008; Shimada et al., 2010), or a genetic vector, which after entering the cell causes expression of the marker in it (Nakatomi et al., 2002; Hou et al., 2008; Lai et al., 2008; Magnusson et al., 2014; Duan et al., 2015).

For labeling brain cells, such stains as 1,1′-dioctadecyl-6,6′-di- (4-sulfophenyl)-3,3,3′,3′ tetramethylindocarbocyanine (DiI), and fluorescent latex microspheres are used. For detection of the labeled cells, fluorescent microscopy is used (Nakatomi et al., 2002; Jin et al., 2003; Zhao et al., 2003; Ramaswamy et al., 2005; Zhang et al., 2005; Faiz et al., 2008; Hou et al., 2008; Shimada et al., 2010). However, the major drawback of this approach is that a cell gets a very limited amount of the label, which can then be lost either due to cell metabolism, or via cell divisions, if labeled cells are proliferating as in the case of NPCs. In this regard, a genetic vector injection is a more appropriate method because even one copy of a reporter gene entering the cell may be enough to cause a stable synthesis of labeling substance in it. Moreover, the application of such genetic vectors as retrovirus or lentivirus, which integrate into the host cell genome, ensures the transmission of the reporter gene to all offspring of the infected cell: this is an advantage for the staining of proliferating cells, for example, NPCs.

Genetic vectors for *in vivo* cells tracing encode many different reporter genes that allow for the identification of targeted cells. These include the green fluorescent protein (GFP) gene, the yellow fluorescent protein (YFP) gene, the DSRed, mCherry, and mKate for detection using fluorescence microscopy; the alkaline phosphatase (AP) gene for immunodetection; the galactosidase (GLA) gene for histochemical detection using transmitted light microscopy (Nakatomi et al., 2002; Goings et al., 2004; Ohab et al., 2006; Kolb et al., 2007; Yang et al., 2007, 2008; Hou et al., 2008; Lai et al., 2008; Liu et al., 2009; Li B. et al., 2010; Ohira et al., 2010; Yoshikawa et al., 2010; Grade et al., 2013; Saha et al., 2013; Magnusson et al., 2014; Müller-Taubenberger and Ishikawa-Ankerhold, 2014; Vandeputte et al., 2014; Duan et al., 2015; de Jong et al., 2017).

Another important question concerns promoters that drive reporter gene expression in vectors for NPCs labeling. The use of general promoters (CMV-promoter, CAG-promoter) in the construct results in non-specific expression of the reporter gene in the cells that have been successfully infected with a genetic vector (Grade et al., 2013; Saha et al., 2013; Magnusson et al., 2014; Duan et al., 2015). Nevertheless, this promoter-related non-specificity may at least in part be compensated by the use of viral serotypes that preferentially target specific cell types (Aschauer et al., 2013), as well as by the use of retroviruses that transduce specifically mitotic cells (Roe et al., 1993). Non-specific expression is in fact an advantage, if the aim of investigation is to trace the cells’ fate throughout their differentiation – when changes in expression of many cell type-specific genes occur. The use of cell type-specific promoter drives expression of the reporter gene in a particular group of cells. It can be a promoter of the gene, the expression of which is a reliable indicator of a certain cell group; for example, DCX – a protein of young neurons, Nestin – of NSCs, GFAP – of NSCs and astroglia. This approach has only a limited potential for studying neurogenesis as it does not allow for the detection of neural cells at different stages of their development.

Interestingly, there is still at least one work in which this approach was applied to investigate the origin of new neurons in the brain. Duan et al. (2015) injected a plasmid carrying the GFP gene under the control of the GFAP gene promoter into the striatum of mice and showed that after ischemia new striatal neurons express the GFP-reporter, despite the lack of GFAP gene expression. The authors concluded that these new neurons in the striatum stem from local GFAP-expressing astrocytes. However, since it remains unclear what could be the cause of GFP expression in these new neurons, the results of the research cannot be unambiguously interpreted.

A completely different approach involves the use of transgenic animals already carrying a reporter gene in their cells. As a rule, the reporter gene is initially inactive because of an insertion containing a stop signal. It can then be restored (the restoration mechanisms are described below), thus facilitating the reconstruction of the reporter gene in the cells residing in a specific brain region at a specific time point and of a specific phenotype. Later, the cells where the reporter gene was restored and their offspring can be detected (Li L. et al., 2010; Bi et al., 2011; Zhang et al., 2011; Magnusson et al., 2014; Faiz et al., 2015).

The reporter gene can be restored by the use of site-specific Cre-recombinase that removes an insertion from the reporter gene (Magnusson et al., 2014; Nato et al., 2015). A specific variant of this recombinase is a CreERT2 recombinase that requires the presence of tamoxifen for activity (Li L. et al., 2010; Zhang et al., 2011; Faiz et al., 2015). Tamoxifen can be either administered intraperitoneally (Li L. et al., 2010; Zhang et al., 2011) or added to daily ration (Faiz et al., 2015). The gene of Cre-recombinase recombinase can be initially present in the animal genome, together with the reporter gene (Li L. et al., 2010; Zhang et al., 2011; Faiz et al., 2015). Alternatively, it can be introduced into the animal brain via vector injection (Magnusson et al., 2014). Additionally, to achieve cell-type specificity of the reporter gene restoration, the recombinase gene can be placed under the control of a cell-specific promoter, for example, the promoter of the GFAP gene (Magnusson et al., 2014; Nato et al., 2015). As a result of these manipulations, reporter gene restoration occurs in a particular brain region, or at least at a specific time point, and in the cells having a particular phenotype.

Lastly, Kreuzberg et al. (2010) used animals carrying the reporter gene under the control of a promoter of the 5HT3A gene, which is active in all, or the majority, of neuroblasts originating from the SVZ, as well as in some mature interneurons. In combination with BrdU administration, it allows the distinguishing of SVZ-derived newborn cells.

Another principally new technique to have been recently developed is the Brainbow strategy (Livet et al., 2007) which aims to identify single cells of one type and trace their connections and lineages. The strategy is based on Cre-Lox recombination that allows the Brainbow transgene to cause expression of three different fluorescent proteins in random combinations and obtain multicolor micrographs of brain sections. Recently, this approach has been successfully applied to trace lineages of NSCs and NPCs in the DG and SVZ for 6 months (Gomez-Nicola et al., 2014). Obviously, this technique has huge potential since it helps distinguish ramified processes of neighboring cells and thereby trace their lineage. However, we believe that the advantages of the Brainbow technique are also its limitations because multicolor single-cell labeling cannot be combined with another type of fluorescent labeling.

Thus, local *in vivo* cell labeling is the most reliable method for establishing the origin of new neurons in brain regions. Rapid development in genetic engineering led to widespread using of plasmid libraries designed for new viral vector production. To increase efficiency of the use of libraries, a novel tool of viral barcoding was suggested (Davidsson et al., 2016). The method of *in vivo* cell labeling is often used together with detection of labeled cells in fixed brain sections. Additionally, this method facilitates observation of the migration of labeled neuroblasts in real time, as described in the section “Real-Time Observation of NPCs Migration.”

### Suppression of NPC Production and Migration

In this section we describe approaches that diminish or exclude any participation of one brain region in the replenishment of neurons in another region. Two methods are applicable here: either locally suppressing the production of new NPCs in one region or preventing the migration of NPCs from one region to another.

Inhibition of neurogenesis in a particular brain region can be achieved by local injection of the antimitotic agent Ara-C, which kills cycling cells. In a number of studies, Ara-C was administered into the lateral ventricle. Thus the role of SVZ neurogenesis in the addition of new neurons in the damaged cortex was examined (Leker et al., 2007; Li B. et al., 2010; Yoshikawa et al., 2010; Faiz et al., 2015). In some works, migration from the SVZ toward the cortex was prevented by surgical separation (Shapiro et al., 2009; Ahmed et al., 2012).

However, it should be mentioned that some issues have complicated the interpretation of the results obtained with the use of these approaches. In particular, antimitotic drugs can diffuse in the brain tissue from the region of injection to remote brain areas. Thus, spatial specificity of antimitotic effect should be carefully verified. Additionally, both local antimitotic drug administration and surgical isolation of brain areas are highly invasive approaches, potentially causing unwanted side effects.

Almost all the above-mentioned studies have demonstrated that the suppression of proliferation in, or migration from, one region affects the number of new neurons in the other; therefore, the results of these experiments cannot be unequivocally interpreted (Leker et al., 2007; Shapiro et al., 2009; Li B. et al., 2010; Yoshikawa et al., 2010; Faiz et al., 2015).

### Real-Time Observation of NPCs Migration

In the last few decades, new methods for longitudinal *in vivo* tracking of NPCs have been devised, while some existing ones have been considerably improved. One of those more recently developed approaches involves MRI-based labeling of migrating NPCs, while others are based on fluorescence or bioluminescence detection.

The most common MRI-based labeling technique uses iron oxide particles as MRI-negative contrast agent visible in T2-weighted images (Norman et al., 1992; Bulte et al., 1999). Several efforts have been made to visualize NPCs by injecting iron oxide nanoparticles directly into the lateral ventricles or the SVZ (Shapiro et al., 2006; Panizzo et al., 2009; Nieman et al., 2010; Sumner et al., 2010; Vreys et al., 2010). However, this method has several significant limitations: cells can release iron oxide particles that can then be absorbed by non-proliferating cells, including macrophages, which are present at the site of damage, thus indicating low specificity of the method; furthermore, neuroblasts can die before reaching the target regions (Winner et al., 2002). Consequently, MRI detection by iron-based nanoparticles does not provide precise information about the state, location and survival of new neurons.

Attempts to overcome low specificity of paramagnetic nanoparticles have been made in a number of works (Elvira et al., 2012; Zhong et al., 2015; Zhang et al., 2016), in which the animal brain was injected with paramagnetic particles conjugated with antibodies to specific cell-surface antigens of NPCs. Another type of cell labeling for MRI is based on reporter genes coding paramagnetic proteins, mainly ferritin (Iordanova and Ahrens, 2012; Vande Velde et al., 2012). This method, though less liable to imaging artifacts compared to iron oxide labeling, shows relatively low MR signal intensity from ferritin overexpression (Naumova et al., 2014). Thus, the reporter gene design and MRI protocols need improving. A similar methodological approach utilizing specific reporter genes was applied for NPC visualization in other modalities. Introduction of the luciferase gene allowed the tracing of NPCs using bioluminescence detection (Reumers et al., 2008; Vandeputte et al., 2014).

Multiple works at different levels of observation were performed with the use of fluorescence. Neuroblast migration dynamics was directly observed on acute brain slices using time-lapse microscopy (Landecker, 2009). In these works, migrating cells are also labeled either by prior administration of fluorescent particles (Zhang et al., 2007) or a vector with a reporter gene (Inta et al., 2008; Kojima et al., 2010; Le Magueresse et al., 2012; Grade et al., 2013) into the animal brain, or by using transgenic animals carrying the reporter gene under the control of a neuron-specific promoter (Dayer et al., 2008; Zhang et al., 2009). The advantage of studies on brain sections, when compared to research into the whole brain, is greater resolution and the possibility of a detailed estimation of particular cells’ behavior. The limitation of this method is the short lifespan of a brain slice – usually studies last up to one day. However, the living whole brain is preferred to long-living brain slices for observations at the molecular level.

The most promising strategy is a recently developed technique of *in vivo* cell tracking with intravital microscopy through a cranial window. Two-photon microscopy is a further improvement of confocal laser scanning microscopy due to its deeper tissue penetration, efficient light detection, and reduced photobleaching (Denk et al., 1990). These benefits allowed for the combination of this technique with intravital observation of living neural cells that had been previously labeled using reporter genes of fluorescent proteins. Recently this technique has been used for the investigation of hippocampal neurogenesis in the DG (Pilz et al., 2016).

Table 1 summarizes the advantages and limitations of the methods described in this section. From our point of view, the methods for local cell labeling and the methods for direct observation of cells, described in the sections “Determining the Place/Time of NPCs Production by Chemical and Genetic Labeling” and “Real-Time Observation of NPCs Migration,” are the most promising, informative, and precise.

**Table 1:** Methods for estimation of NPCs migration in the brain.

Method	Description of the method	Advantages	Limitations
Estimation of neuroblasts migration patterns in fixed brain slices.	In fixed brain slices signs of neuroblasts migration are observed: distribution of cells from the supposed place of origin to the supposed destination; orientation of chains of cells and cell processes in the direction toward the destination.	It is a non-invasive and simple research method that facilitates the estimation of cell migration in the brain at the time of animal euthanasia.	This approach facilitates the estimation of the direction of migration only of the cells migrating at the time of animal euthanasia. Furthermore, although the distribution of neuroblasts in the fixed brain slice describes the general direction of migration, it fails to prove the origins and direction of migration of each cell.
Local administration of antimitotic agents.	The Ara-C antimitotic agent is administered into a particular brain region to suppress neurogenesis in it.	The method facilitates the reduction or exclusion of any participation of a brain region in replenishment with neurons in another region.	The method is invasive and potentially causes adverse effects. The results of such experiments can be unequivocally interpreted only in the absence of any effects, i.e., when killing proliferating cells in one region doesn’t affect the number of new neurons in the other.
			Moreover, antimitotic drugs can diffuse in brain tissue from the region of injection to the remote brain areas. Thus, spatial specificity of antimitotic effect should be carefully verified.
Surgical separation of brain regions.	Surgical separation prevents migration from one brain region into another.	This method facilitates the exclusion of any participation of a brain region in replenishment with neurons in another region.	The method is invasive, and potentially causes adverse effects.
Local labeling of cells in the brain.	Local administration of stains or genetic vectors carrying the reporter gene. Alternatively, transgenic animals can be administered with special agents that induce expression of a reporter gene in them (tamoxifen or a genetic vector with the recombinase gene can serve as an inductor).	This method helps prove the origin of a particular neuron from a particular brain region. Moreover, the method is suitable for direct observation of cell migration.	The method is invasive, and potentially causes adverse effects.
Direct observation of cell migration in live brain slices.	Preliminary *in vivo* staining of neuroblasts, obtaining of live brain slices and detection.	The method facilitates a detailed observation of individual cell behavior.	The conditions in the live brain slice cannot be equal to the conditions in the live animal brain: cutting the brain into slices results in damage to many cells, and isolating a slice from the whole brain removes potential influences from other cells in the whole brain. Moreover, brain slices remain viable for quite a short time (usually studies last up to one day), which is not enough to observe the long-term fate of migrated cells.
Direct observation of *in vivo* cell migration.	Preliminary *in vivo* staining of neuroblasts and subsequent bioluminescent or MRI detection. Alternatively, two-photon microscopy through a cranial window.	The method facilitates the tracing of long-term cell migration in the live brain.	In general, the method has a low-resolution capacity, as it facilitates the observation of the migration of only large cell groups. However, a recently developed technique of intravital microscopy through a cranial window, in combination with two-photon microscopy, facilitates the tracing of individual cells deep within the brain tissue. Still, cranial window is a highly invasive approach.


## Early Postnatal and Adult Neurogenesis in the Normal Brain

In this section we summarize the data accumulated to date about the origins of NPCs and their migration pathways in the normal mammalian brain during the early postnatal and adult periods.

### The Ventricular-Subventricular Zone

The V-SVZ is the main neurogenic niche and the major source of new neurons in the postnatal brain, both in the early postnatal period and throughout adulthood. This is a place where NSCs reside and NPCs originate. Many authors have recently used the general term, V-SVZ, to describe several regions located in the walls of the lateral ventricles (Lim and Alvarez-Buylla, 2016). The SVZ, which stretches along the lateral wall of the lateral ventricles, is a major focus of studies (Doetsch et al., 1997, 1999; Bernier et al., 2000; Gage, 2000; Kornack and Rakic, 2001; Pencea et al., 2001a; Temple, 2001; Alvarez-Buylla et al., 2002; Doetsch, 2003; Ming and Song, 2005; Duan et al., 2008). Additionally, there are data on NSCs in a ventral extension of the lateral ventricle (Zhao et al., 2003) and in the PPV region (Nakatomi et al., 2002; Bull and Bartlett, 2005; Abdipranoto-Cowley et al., 2009; Saha et al., 2013).

Neural stem cells in the V-SVZ, the so-called type B1 cells, have many characteristics of parenchymal astrocytes because they express GFAP, glutamate aspartate transporter (GLAST), and brain lipid-binding protein (BLBP) (Lim and Alvarez-Buylla, 2016). However, unlike parenchymal astrocytes, type B1 cells have a direct contact with the ventricle. A subset of these cells is quiescent NSCs, whereas others are mitotically active (Llorens-Bobadilla et al., 2015). In the activated state, type B1 cells also express Nestin and produce transit-amplifying precursors (type C cells) that produce NPCs (type A cells) expressing the markers of immature neurons (Lim and Alvarez-Buylla, 2016). The NPCs that originate in the V-SVZ migrate to other brain regions where they finally differentiate into neurons and may integrate into local neural networks (Lledo and Saghatelyan, 2005; Sakamoto et al., 2014).

In the early postnatal brain, several migratory pathways stretching from the V-SVZ to other brain regions have been described. Inta et al. (2008) showed in fixed and cultivated mouse brain slices that in the first weeks of life, neuroblasts travel along four different pathways – rostral, ventral, external, and dorsal – from the lateral ventricles. The rostral migratory stream (RMS) flows from the SVZ to the OB; the ventral stream flows from the SVZ to the striatum and the nucleus accumbens; the external migratory pathway emerges from the anterior parts of the SVZ and extends along the external capsule toward the latero-dorsal regions; the dorsal stream flows from the PPV along the superior border of the hippocampus toward the occipital cortex. Other authors also report on the massive migration of neuroblasts from the SVZ as well as from the RMS via the corpus callosum to the cortex (Dayer et al., 2008; Le Magueresse et al., 2012). In the cortex, migrating neuroblasts mainly reach the lower layers (Dayer et al., 2008; Inta et al., 2008; Le Magueresse et al., 2012), but can be also found in the upper layers (Dayer et al., 2008; Inta et al., 2008).

De Marchis et al. (2004) injected CellTracker Green into the SVZ of newborn mice and showed three migratory pathways for neuroblasts originating in the SVZ: primarily, the RMS and two additional pathways – ventral migratory mass (VMM) and ventrocaudal migratory stream (VMS). Within the VMM, neuroblasts migrate from the SVZ to the basal forebrain and populate the islands of Calleja, while the VMS deviates from the anterior part of the RMS to flow in the ventrocaudal direction, when neuroblasts reach the pyramidal layer of the olfactory tubercle.

In the adult brain, the V-SVZ remains the main zone of neurogenesis. However, its intensity decreases soon after birth and the majority of pathways become largely quiescent (Fuentealba et al., 2015). The exception is NPC migration from the SVZ to the OB, which remains active in adulthood, at least in rodents (Lim and Alvarez-Buylla, 2016). Cells migrate in chains and form an RMS stretching from the SVZ to the OB. In the OB, neuroblasts then migrate radially and turn into mature neurons in the granular and periglomerular layers (Sun et al., 2011).

The migration from the V-SVZ to other brain regions substantially decreases compared with the early postnatal period, and a range of works have confirmed this. De Marchis et al. (2004) found that migration via the VMS to the olfactory tubercle, which was observed in neonatal mice, is less intense during adulthood, whilst migration within the VMM stops completely. Several other studies report on the occurrence of neuroblasts and new mature neurons in different areas of the adult mouse and rat brains, including the striatum, nucleus accumbens, ventral septum, corpus callosum, olfactory tubercle, anterior olfactory nuclei, tenia tecta, islands of Calleja, amygdala and lateral entorhinal cortex (Dayer et al., 2005; Sundholm-Peters et al., 2005; Shapiro et al., 2009). The origin of these neurons has not been established. However, the authors posit that they are produced in the SVZ (Dayer et al., 2005; Sundholm-Peters et al., 2005; Shapiro et al., 2009). Zhao et al. (2003) labeled NPCs localizing near the lateral ventricles with DiI injection into the lateral ventricle, and demonstrated that neuroblasts migrate from a ventral extension of the lateral ventricle to the substantia nigra, where they differentiate into dopaminergic neurons. Nakatomi et al. (2002) administered DiI or a genetic vector with a GFP gene into the lateral ventricle of a normal animal, and showed negligible migration of stained neuroblasts from the PPV toward CA1 of the hippocampus.

Despite the fact that these pathways remain largely quiescent (Fuentealba et al., 2015), they might be important for response to injury. Multiple studies on the injured brain showed significant intensification of NPC migration in these conditions (Arvidsson et al., 2002; Nakatomi et al., 2002; Thored et al., 2006).

In humans, the amount of neuroblasts migrating to the OB drops rapidly in the early postnatal period. Spalding et al. (2013) using the ^14^C technique showed that in the OB, adult neurogenesis can be negligible. As in animal models, neurogenesis in the SVZ in humans declines over a lifetime (Dennis et al., 2016).

### The Hippocampus

The DG of the hippocampus ranks second after the V-SVZ where proliferation of NPCs continues throughout the whole life of animals. According to the classical point of view, the dentate neuroepithelium gives rise to both granular neurons and NSCs in the prenatal period (Gonçalves et al., 2016). However, recent studies of Li et al. (2013) showed that the NSCs in the DG may originate from the population in the most ventral part of the hippocampus, close to the lateral ventricle. These cells migrate to the DG at the late stage of gestation and become the source of NSCs of the SGZ in adulthood. Proliferation in the DG is limited to the SGZ from the second postnatal week (Urbán and Guillemot, 2014). Radial glia-like cells (RGL), also called type 1 cells that express GFAP, Nestin, and SOX2, are considered as genuine NSCs in the SGZ (Gonçalves et al., 2016). Most of these cells remain quiescent and eventually divide (Encinas et al., 2011).

Unlike the SVZ (Obernier et al., 2018), neurogenic potential of NSCs in the SGZ is highly debated. Bonaguidi et al. (2011) showed self-renewal properties and multipotent capacity of RGL cells, using cloning analysis. At the same time, the studies of Encinas and Enikolopov, based on the Nestin-GFP transgenic mice (Mignone et al., 2016), showed restriction of the NSC pool and its inability to self-renew. Moreover, the authors, using multiple S-phase labeling, observed that RGL cells in the SGZ make 2-3 asymmetric divisions and then lose their RGL morphology. Further they move to the hilus where they start expressing S100ß (a marker of mature astrocytes) and continue to express GFAP but not Nestin (Encinas et al., 2011). The number of RGL cells dramatically drops with age (Gage, 2000; Encinas et al., 2011; Ming and Song, 2011; Bergami et al., 2015).

Asymmetric division gives rise to bipolar neural progenitors that differentiate into neuroblasts after several divisions. During their maturation these NPCs, unlike NPCs from the V-SVZ, migrate short distances – from the subgranular to the granular layer of the DG – and become mature glutamatergic granular neurons (Gage, 2000; Alvarez-Buylla et al., 2001; Temple, 2001; Doetsch, 2003; Ming and Song, 2005; Duan et al., 2008; Imayoshi et al., 2008).

In adult humans, hippocampal neurogenesis in the DG is more prominent than in the OB (Gage, 2000; Spalding et al., 2013; Jessberger and Gage, 2014). Spalding et al. (2013) showed two different types of neuronal populations in the human hippocampus, one of which is renewed continuously, unlike the other. The renewing cell population accounts for approximately one third of the hippocampal neurons and exceeds similar cell population in rodents (Snyder and Cameron, 2012). In humans, as in rodents, hippocampal neurogenesis declines over the years (Ihunwo et al., 2016). It is worth noting that despite the large number of research works, inconsistency still exists concerning the abundance of adult human hippocampal neurogenesis. For example, two recent studies have provided quite contradictory results. Moreno-Jiménez et al. (2019) found thousands of cells of immature neuronal phenotype within the DG of healthy adult subjects, aged between 43 and 87. In sharp contrast, Sorrells et al. (2018) report that hippocampal neurogenesis declines in children and no immature neurons are found within the DG in adult subjects. Moreno-Jiménez et al. (2019) along with Kempermann et al. (2018) consider that such discrepancies may stem from the differences in the details of post-mortem tissue processing, and conclude that the level of human hippocampal neurogenesis may in fact be underestimated in some studies due to the loss of the detectable immature neuronal markers in post-mortem brain tissues. Thus, a consensus about the level of adult human hippocampal neurogenesis is yet to be achieved.

### The Cortex

Neurogenic potential of the cerebral cortex in physiological conditions is considerably smaller in comparison to the V-SVZ and to the SGZ of the DG.

The majority of cortical neurons and glial cells originate from the radial glia stem cells that located along lateral ventricles. These cells generate cortical neurons and direct their migration to the cortical layers at the embryonic stage (Rakic et al., 2009). This process is almost complete prior birth. However, the mouse cortex retains its neurogenic potential for a short period after birth. For almost 10 days after birth, there exist in the mouse cortex multipotent astrocytic stem cells, which, having been translated into cell culture, form neurospheres and differentiate into neurons, astrocytes and oligodendrocytes (Laywell et al., 2000; Ahmed et al., 2012). From day 10 neurogenesis in the cortex stops; however, traumatic injury of the cortex at day 15 causes its restoration (Ahmed et al., 2012).

Almost no neurogenesis occurs in the cortex of the normal adult brain in rodents, but systematic analysis shows that small numbers of NeuN/BrdU double positive new neurons still continue to be generated there (Dayer et al., 2005). Their origin has yet to be established, but the authors posit that these neurons might be produced from local precursors. Interestingly, these new neurons seem to be generated not from DCX-expressing cells but, rather, from those that express NG2, a protein that is usually associated with oligodendrogenesis. There were no DCX-expressing cells in the cortex, but instead there were proliferating (BrdU-labeled) NG2-positive cells. Moreover, some NeuN/BrdU positive cells also co-expressed NG2, suggesting that NG2-precursors are the source of new neurons in the cortex. Given that DCX is a marker of migrating neuroblasts, the authors concluded that the observed cortical DCX-negative progenitors are of local origin (Dayer et al., 2005). Gould et al. (2002) similarly showed rare cells incorporating BrdU and co-expressing the neuronal markers NeuN and TuJ1 in the anterior rat neocortex. It remains unclear whether these cells are of local origin or they arise from the SVZ. At the same time, some other studies failed to discover any newly generated neurons in the adult rodent cortex (Ehninger and Kempermann, 2003; Madsen et al., 2005). However, analysis performed in the review by Cameron and Dayer (2008) suggests that the causes for such negative results may stem from an extremely low level of cortical neurogenesis and the small diameter of their nuclei.

A number of studies in primates and humans mainly report on the absence or a low level of adult neurogenesis in the cerebral cortex. Longitudinal observations by Gould et al. (2002) revealed a small number of cells that co-expressed the neuronal markers NeuN and TuJ1 with BrdU. The authors concluded that these *de novo* generated neurons have transient existence because their number declines after 9 weeks of observation. The origin of these cells has not been studied in this work. In humans, Eriksson’s studies (Eriksson et al., 1998) showed a non-neuronal phenotype of BrdU-labeled cells in the cortex. An extremely sensitive analysis, which included both BrdU labeling and ^14^C incorporation, estimated the exchange rate of neurons in the adult human neocortex at 1/1,000 neurons every fifth year (Bhardwaj et al., 2006).

For several decades, the piriform cortex has attracted significant attention due to its ability to express the markers of immature neurons DCX and PSA-NCAM, which was revealed in most mammalian species in adulthood. Nevertheless, numerous attempts to achieve evidence of neurogenesis in this area using BrdU labeling failed. It was concluded that the expression of DCX and PSA-NCAM could be explained by this particular population of neurons retaining its structural plasticity (Nacher and Bonfanti, 2015).

### The Striatum

The adult striatum, at least in some mammalian species, also retains the potential to generate new neurons. Ontogenically, the striatum originates from the ganglionic eminence located in the ventral part of the developing telencephalon. The most active striatal neurogenesis, which proceeds in two phases that result in a different functionality of striatal neurons, almost ends with birth (Pauly et al., 2012). In adults the striatum consists of about 95% GABAergic medium spiny neurons and less than 5% aspiny interneurons (Tepper and Bolam, 2004; Pauly et al., 2012).

Unlike the cerebral cortex, there is significantly more evidence of the generation of new neurons in the adult striatum under physiological conditions. Several works did not reveal new neurons in the unlesioned striatum (Pencea et al., 2001b; Teramoto et al., 2003; Luzzati et al., 2011). Nevertheless, some studies clearly demonstrated that neuroblasts and new neurons continued to arise in the striatum of adult rats (Dayer et al., 2005), rabbits (Luzzati et al., 2006), guinea pigs (Luzzati et al., 2014) and humans (Ernst et al., 2014). However, adult-born new neurons were not found in mice in physiological conditions (Nato et al., 2015), suggesting a potentially important difference between the species. In humans, unlike in other species, Ernst et al. (2014) showed substantial neurogenesis in the striatum: a subpopulation of interneurons, mainly expressing the marker calretinin, is exchanged at a rate of 2.7% per year in adulthood.

The origin of NPCs in the adult striatum has been intensively studied. Some works have largely clarified the question concerning “local vs. SVZ” source of these neurons. Luzzati et al. (2006), analyzing spatial distribution of proliferating NPC clusters in rabbits, have demonstrated that new neurons in the striatum, at least partially, are of local origin. The authors also showed that NPCs differentiate into mature neurons; however, only a small amount of them survive during maturation. Similarly, NSCs of astroglial nature, like NSCs in the SVZ and DG, were found in the external capsule and lateral striatum of juvenile guinea pigs (Luzzati et al., 2014). These cells proliferate and give rise to new neurons that have existed transiently and have not contributed to the population of mature functional neurons. At the same time, they expressed Sp8, a transcription factor associated with neuroblasts migrating to the OB, and showed tropism for white matter tracts. Collectively, these studies suggest that the striatum, at least in rabbits and guinea pigs, is able to generate new neurons but that their survival is negligible.

Various pathologic changes and injuries strongly affect neurogenesis and migration of NPCs in the adult brain. Though the V-SVZ is still considered the main source of new neurons, migration pathways and destinations of NPCs can change depending on lesion area. At the same time, there is much evidence that new neurons can originate from different sources outside the known and proven neurogenic zones. In the next section we consider several works investigating these changes.

## Neurogenesis in the Injured Brain

In this section we describe changes in proliferation and migration of NPCs in the injured brain. In general, injury can stimulate V-SVZ neurogenesis accompanied by the migration of V-SVZ-derived neuroblasts to the site of injury, as well as local generation of new neuroblasts near the damage area. In the latter case, new neuronal progenitors are found to arise from local astrocytes, which gain NSCs properties. The significance of SVZ-derived versus local progenitors is a highly debated question. Table 2 summarizes the research described in the following sections.

**Table 2:** Data on the origins of new neurons in the damaged brain.

Model of brain injury	Brain region						References
		
	CA1 region of the hippocampus		Striatum		Cortex		
				
	Resident	Migrating from the V-SVZ	Resident	Migrating from the V-SVZ	Resident	Migrating from the V-SVZ	
Global ischemia		Yes					Nakatomi et al., 2002
		Yes					Oya et al., 2009
MCAO			No	Yes			Yamashita et al., 2006
			Yes				Magnusson et al., 2014; Duan et al., 2015
				Yes			Arvidsson et al., 2002; Zhang et al., 2004, 2007, 2009, 2011; Thored et al., 2006; Hou et al., 2008; Liu et al., 2009; Kojima et al., 2010; Li L. et al., 2010; Nakaguchi et al., 2012; Grade et al., 2013; Kazanis et al., 2013
Neonatal ischemia/hypoxia				Yes			Yang and Levison, 2006; Yang et al., 2008
Global ischemia				Yes			Yoshikawa et al., 2010
Hemorrhagic stroke				Yes			Yan et al., 2009a
MCAO				Yes		Yes	Parent et al., 2002; Jin et al., 2003; Zhang et al., 2005; Yan et al., 2007
Global ischemia					Yes	Yes	Ohira et al., 2010
					Yes		Fukuzaki et al., 2015
Neonatal hypoxia					Yes		Bi et al., 2011
MCAO					Yes	No	Kuge et al., 2009
Chemical injury of the cortex					No	Yes	Faiz et al., 2015
						Yes	Magavi et al., 2000; Chen et al., 2004; Faiz et al., 2008
Global ischemia						Yes	Li et al., 2011
MCAO						Yes	Leker et al., 2007; Wang et al., 2007; Lai et al., 2008; Li B. et al., 2010; Kreuzberg et al., 2010; Moraga et al., 2014; Palma-Tortosa et al., 2017; Pradillo et al., 2017
MCAO+global ischemia						Yes	Ohab et al., 2006; Song et al., 2017; Tseng et al., 2018
Photothrombotic stroke (cortex)						Yes	Osman et al., 2011; Vandeputte et al., 2014
Thermocoagulation of pial vessels (cortex)						Yes	Gotts and Chesselet, 2005
Cortical devascularization						Yes	Kolb et al., 2007; Wan et al., 2016
Neonatal ischemia/hypoxia						Yes	Yang et al., 2007
Mechanical injury of the cortex						Yes	Goings et al., 2004; Ramaswamy et al., 2005; Saha et al., 2013; Yi et al., 2013


### New Neurons in the Damaged Hippocampus – Resident and Migrating From the PPV Region

As has already been mentioned, under physiological conditions significant neurogenesis occurs in the DG of the hippocampus. Under ischemic conditions, mainly in the model of global ischemia, the number of neuroblasts in the DG can grow (Schmidt and Reymann, 2002; Bendel et al., 2005; Wojcik et al., 2009; Khodanovich et al., 2016, 2018a).

Additionally, after global ischemia new neurons are detected in the CA1 region of the hippocampus, where in these conditions a massive death of pyramidal neurons occurs (Nakatomi et al., 2002; Schmidt and Reymann, 2002; Daval et al., 2004; Bendel et al., 2005; Oya et al., 2009; Wojcik et al., 2009). The PPV is probably the major source of these new neurons in CA1. This supposition is lent credence by the work of Nakatomi et al. (2002) who, having labeled the cells from the PPV by intraventricular administration of DiI or of a vector with a GFP gene, proved that under total ischemic conditions neuroblasts migrate from the PPV. Moreover, the authors showed that a small number of neuroblasts from the PPV also migrate to the DG. Some authors revealed post-ischemic distribution of neuroblasts from the PPV to CA1 in fixed slices (Oya et al., 2009; Khodanovich et al., 2018b). Furthermore, it is likely that the same migration was being reported in the work by Daval et al. (2004), wherein the authors discovered an increase, after neonatal hypoxia, in the number of neuroblasts in the SVZ and the PPV, where labeled cells formed a migrating chain toward the periventricular region located above the hippocampus. Many works that have demonstrated the appearance of young neurons in CA1 after ischemia still provide no information about their origins (Schmidt and Reymann, 2002; Bendel et al., 2005; Wojcik et al., 2009; Niv et al., 2012; Ortega et al., 2013). Bendel et al. (2005) emphasized that after ischemia NPCs proliferation increased both in the PPV and the SGZ of the DG, and both these regions could be the source of new neurons in CA1.

Thus, these data testify that new neurons in the damaged CA1 region originate from the PPV. However, there is no proof that resident NPCs cannot occur in this region after an injury, because this possibility has not been specifically examined.

### New Neurons in the Damaged Striatum – Resident and Migrating From the SVZ

In the damaged striatum, many young neurons can be detected, as has been shown on models of ischemia (Jin et al., 2003; Yamashita et al., 2006; Hou et al., 2008; Kojima et al., 2010; Grade et al., 2013; Magnusson et al., 2014) and neonatal ischemia-hypoxia (Yang and Levison, 2006; Yang et al., 2008).

Some works do not specify the origins of these cells (Kobayashi et al., 2006; Lee et al., 2006; Zhang et al., 2006, 2010; Masuda et al., 2007; Yoo et al., 2008; Cui et al., 2009; Yan et al., 2009a; Shimada et al., 2010; Li et al., 2011; Ortega et al., 2013; Cheyuo et al., 2015; Fujioka et al., 2017; Wang et al., 2017). However, some proved their origins from the SVZ using different methods by:

- labeling cells in the SVZ (Jin et al., 2003; Zhang et al., 2005, 2007, 2011; Yamashita et al., 2006; Hou et al., 2008; Yang et al., 2008; Liu et al., 2009; Kojima et al., 2010; Li L. et al., 2010; Yoshikawa et al., 2010; Grade et al., 2013);
- direct observation of migration in living brain slices (Zhang et al., 2007, 2009; Kojima et al., 2010; Grade et al., 2013);
- intraventricular administration of the antimitotic agent Ara-C (Yoshikawa et al., 2010);
- or estimation of neuroblasts distribution in fixed brain slices (Arvidsson et al., 2002; Parent et al., 2002; Jin et al., 2003; Zhang et al., 2004, 2005, 2007, 2009, 2011; Thored et al., 2006; Yang and Levison, 2006; Yan et al., 2007, 2009b; Yang et al., 2008; Liu et al., 2009; Li L. et al., 2010; Yoshikawa et al., 2010; Nakaguchi et al., 2012; Kazanis et al., 2013).

Additionally, data provided by Yamashita et al. (2006) suggest that SVZ-derived NPCs can be the main source of new neurons in the damaged striatum after transient MCAO, with no, or minor, participation of local striatal progenitors. For labeling cells that originate from the striatum, a genetic vector carrying the GFP gene was injected into the striatum. As a result, GFP expression was induced in some striatal cells (in the majority of cells near the injection site), but not in the SVZ. Then, ischemia was induced, and none of the detected DCX-positive neuroblasts in the damaged striatum expressed GFP. However, given that only a subset of striatal cells was infected with a GFP-expressing vector, the generation of new neurons from some unlabeled striatal progenitors cannot be entirely excluded. At the same time, DCX-positive neuroblasts in the damaged striatum expressed GFP, if the vector was injected into the SVZ. This points to a significant role for SVZ-derived progenitors.

At the same time, three works proved that after lesion, a number of new neurons in the striatum are generated from local precursors (Magnusson et al., 2014; Duan et al., 2015; Nato et al., 2015). Nato et al. (2015) found local neurogenesis in the damaged striatum after excitotoxic lesion. By injecting a vector carrying Cre-recombinase under the control of a GFAP promoter into the striatum of transgenic R26R reporter mice, the authors induced YFP expression in striatal – but not subventricular – astrocytes. After subsequent lesion, about 85% of clustering Ki67 and DCX-positive cells in the damaged striatum co-expressed a YFP reporter, indicating that the majority of lesion-induced neuroblasts are of local origin. Additionally, the induction of a YFP reporter in the SVZ revealed that only a small number of these striatal neuroblasts derived from the SVZ. Similarly, Magnusson et al. (2014) induced the expression of the YFP gene in the striatal astrocytes and their offspring (but without induction of YFP expression in the SVZ or the RMS). After ischemia, which was later induced, many cells that had expressed YFP were also colocalized with DCX and with NeuN. Duan et al. (2015) induced GFP expression in astrocytes of the striatum by injecting a vector carrying a GFP gene under the control of a GFAP promoter. After ischemia they discovered NeuN- and Nestin-positive cells among those that had expressed GFP. The authors concluded that these cells are GFP-positive because they had generated from striatal GFAP-expressing astrocytes. However, it is unclear whether cells are able to retain a detectable amount of GFP in the absence of GFP-gene expression for an any significant length of time. Thus, the exact mechanism of GFP appearance in GFAP-negative neurons and NPCs remains an open question and the results of Duan et al. (2015) are difficult to interpret.

It should be noted here that the majority of the above-mentioned works estimated the role of either the SVZ or only of the striatum in the production of new neurons in the damaged striatum. Only the two works investigated both and obtained contradictory results (Yamashita et al., 2006; Nato et al., 2015). In particular, Yamashita et al. (2006) failed to discover new striatal neurons of local origin at all, whereas Nato et al. (2015) found that the majority of lesion-induced striatal neuroblasts are of local origin. It is unclear whether these contrasting results stem from the difference in the nature of the lesion (transient local ischemia versus chemical lesion), or from some other methodological aspects of the studies. At the same time, the work by Li L. et al. (2010), who labeled the cells originating from the SVZ, showed that labeled cells from the SVZ account for only one third of all neuroblasts in the damaged striatum. However, it remains unclear whether this value is indicative of the actual proportion of subventricular/striatal precursors or of insufficient labeling of precursors in the SVZ. Thus, it remains to be elucidated which plays a more important role in the restoration of the damaged striatum – the SVZ or the striatum.

### New Neurons in the Damaged Cortex – Resident and Migrating From the SVZ/PPV Region

Damage to the cortex of the adult animal brain enhances neurogenesis in it, and this has been shown in different models of brain damage, such as ischemia (Jin et al., 2003; Ohab et al., 2006; Kolb et al., 2007; Kreuzberg et al., 2010; Ohira et al., 2010), neonatal ischemia-hypoxia (Yang et al., 2007), neonatal hypoxia (Bi et al., 2011), and mechanical (Saha et al., 2013), chemical (Faiz et al., 2008, 2015) and thermal (Covey et al., 2010; Ajioka et al., 2015) damage. Some of the above works do not include an investigation into the origins of new neurons in the damaged cortex (Zhang et al., 2006; Yoo et al., 2008; Covey et al., 2010; Ortega et al., 2013; Ajioka et al., 2015).

The majority of works establish their origin from the SVZ or from the PPV. These findings were obtained with the use of different approaches based on:

- distribution pattern of neuroblasts migrating from the SVZ/PPV to the damaged area in fixed brain slices (Magavi et al., 2000; Parent et al., 2002; Jin et al., 2003; Chen et al., 2004; Goings et al., 2004; Gotts and Chesselet, 2005; Ramaswamy et al., 2005; Zhang et al., 2005; Ohab et al., 2006; Kolb et al., 2007; Leker et al., 2007; Wang et al., 2007; Yan et al., 2007; Faiz et al., 2008, 2015; Li et al., 2008, Li B. et al., 2010; Li et al., 2011; Kreuzberg et al., 2010; Osman et al., 2011; Saha et al., 2013; Yi et al., 2013; Moraga et al., 2014; Vandeputte et al., 2014; Wan et al., 2016; Palma-Tortosa et al., 2017; Pradillo et al., 2017; Song et al., 2017; Tseng et al., 2018);
- suppression of cell proliferation in the SVZ by intraventricular administration of Ara-C (Leker et al., 2007; Li B. et al., 2010; Faiz et al., 2015);
- labeling cells from the lateral ventricles (Jin et al., 2003; Goings et al., 2004; Ramaswamy et al., 2005; Zhang et al., 2005; Ohab et al., 2006; Kolb et al., 2007; Yang et al., 2007; Faiz et al., 2008, 2015; Lai et al., 2008; Kreuzberg et al., 2010; Li B. et al., 2010; Saha et al., 2013; Vandeputte et al., 2014);
- and direct observation of migration in the live brain (Vandeputte et al., 2014).

At the same time, some studies do in fact suggest that the production of new neurons can occur in the injured cortex (Gu et al., 2000; Kuge et al., 2009; Ohira et al., 2010; Shimada et al., 2010, 2012; Bi et al., 2011; Nakagomi et al., 2011, 2015; Fukuzaki et al., 2015). Bi et al. (2011) showed by special labeling that, after neonatal hypoxia, cortex astrocytes in neonates gain the properties of NSCs and differentiate into new neurons both in the damaged cortex and after having been translated into cell culture.

Ohira et al. (2010) discovered, in the first layer of the normal animal cortex, proliferating cells that express the GAD67 neuronal marker. Mild ischemia, which was achieved by 10-min cross-clamping of the two major carotid arteries, caused an increase in the number of these cells, their migration into the lower layers of the cortex and differentiation into functionally mature neurons (revealed by expression of the TuJ1, HuC/D, MAP2, c-Fos markers, and electrophysiological activity). A vector with the GFP reporter gene was administered before ischemia either into the cortex or into the SVZ to determine the source of new neurons in the cortex. The cortex appeared to be the main source of new neurons itself, with only a few having migrated to the cortex from the SVZ.

Kuge et al. (2009) report in their work that after transient MCAO new neurons were generated in the cortex. The authors emphasize that they have not detected any patterns of neuroblasts migration from the SVZ via the corpus callosum to the cortex. Therefore, they assume that these new neurons in the cortex are of local origin.

Fukuzaki et al. (2015) described in their work that after 10-min cross-clamping of the two carotid arteries they observed proliferation of GAD67-positive NPCs in the cortex. The fact that these cells localized mainly in the first layer showed that they were local precursors.

Gu et al. (2000) reported in their work that after phototrombotic stroke new neurons (colocalization of bromodeoxyuridine with MAP2 and NeuN) started generating in the damaged cortex 72 h after surgery. The authors concluded that this early appearance of new neurons in the cortex proves that it is very unlikely that they could have originated in the SVZ. At the same time, it is questionable that even local progenitors in the cortex can generate mature NeuN-expressing cortical neurons within 3 days. Thus, the mechanism of new neuron generation in this study remains unclear.

Some works described that after permanent MCAO in adult animals, cortical astrocytes (Buffo et al., 2008; Shimada et al., 2010, 2012), as well as pericytes of leptomeningeal and cortical vessels (Nakagomi et al., 2011, 2015), gained NSC properties: in cell culture they differentiate into neurons, astrocytes and oligodendrocytes. At the same time, in the damaged cortex these precursors exhibited a more limited capacity to differentiate – differentiating only into astrocytes and oligodendrocytes and never into neurons (Buffo et al., 2008; Shimada et al., 2010).

Despite a large number of studies, it is still difficult to get a full picture of neurogenesis in the damaged cortex since the majority of researchers estimated the role of either only the cortex or of the SVZ in the production of new cortical neurons. The work by Ohira et al. (2010) is a rare exception. The authors labeled both cells of the cortex and cells of the SVZ, and demonstrated that after a mild cortical ischemia a larger part of new cortical neurons originated in the cortex, not in the SVZ. At the same time, Faiz et al. (2015) showed that after chemical cortical damage, all of the NPCs that were generated in the cortex had originated from the SVZ. The different results allow us to assume that the role of local and SVZ precursors in the restoration of the cortex can substantially vary depending on the conditions of the experiment, particularly on the type and degree of damage.

### Interhemispheric Migration

In this section we consider several works that describe interhemispheric migration of neuroblasts after cortical injury (Ramaswamy et al., 2005; Wan et al., 2016). The authors discovered that damage to the cortex may result in migration of neuroblasts from one hemisphere to the other. While Wan et al. (2016) reported on migration from the healthy hemisphere to the damaged one, Ramaswamy et al. (2005) discussed migration from damaged to healthy.

Wan et al. (2016) observed that after cortical devascularization, migration occurs not only from the SVZ of the same hemisphere, but also from the contralateral SVZ, to the injured cortex. In fixed brain slices, the authors observed distribution of cells migrating from the contralateral SVZ via the midline, further to the ipsilateral corpus callosum, and finally to the lesion area in the cortex. Some authors report on the enhancement of proliferation in the contralateral SVZ after ischemic or mechanical brain damage but they have not found any signs of migration from the contralateral SVZ to ipsilateral (Leker et al., 2007; Kreuzberg et al., 2010; Li B. et al., 2010; Saha et al., 2013).

Ramaswamy et al. (2005) obtained more unusual results. They discovered that after mechanical damage to the cortex, neuroblasts migrate from the ipsilateral SVZ not only to the lesion area in the cortex, but also to the contralateral (non-damaged) hemisphere. The origin of neuroblasts migrating from the SVZ has been proved by preliminary administration of fluorescent microspheres into the lateral ventricle. The authors hypothesized that migration of neuroblasts to the contralateral hemisphere may functionally compensate for the damage to the ipsilateral cortex, hence the healthy cortex in the contralateral hemisphere may take over the functions of the damaged cortex. The results obtained by the two mentioned groups (Ramaswamy et al., 2005; Wan et al., 2016) are extraordinary, and have not been reported elsewhere. Still, it remains unclear whether migration from the damaged hemisphere to the health one or vice versa occurs only under some specific conditions set by researchers, or is an ordinary process in the injured brain that has just been overlooked by other investigators.

### Functional Relevance of Injury-Induced Neurogenesis

Although this review focuses mainly on the anatomical origin of new neurons appearing in the damaged brain areas, the question about their functional relevance cannot be ignored due to its high importance when seeking out new directions for future therapies. Here we discuss some related issues. For more information about the survival, differentiation and functional integration of new neurons within the injured brain tissue, we direct the reader to the reviews by Lindvall and Kokaia (2015) and Marlier et al. (2015).

Relatively few works have addressed the study of structural and functional integration of new neurons into the existing neural networks after brain injury. Mainly, such integration has been shown in the damaged striatum and cortex.

At least two studies provided evidence that both SVZ-derived and locally generated neurons may successfully integrate into the pre-existing circuit within the damaged cortex. Lai et al. (2008) used lentiviral labeling of SVZ-derived progenitors in combination with patch-clamp recordings on the labeled neurons within the cortex. They showed that after MCAO, new SVZ-derived neurons in the damaged cortex fired the induced and spontaneous action potentials, which suggests that these neurons are synaptically integrated. However, the results of Lai et al. (2008) should be interpreted with consideration for the fact that a lentiviral vector could have infected non-dividing cells in the cortex. Ohira et al. (2010) retrovirally labeled the local progenitors within the cortex, and after global ischemia indirectly examined the functional integration of new neurons by evaluation of the immediate early gene c-Fos expression. They found that c-Fos expression by the new neurons was much higher in the enriched environment when compared with the control conditions, which suggests that the new neurons in the cortex are synaptically active. It was also concluded that the new neurons are GABAergic interneurons.

Several studies showed structural and functional integration of new neurons into the existing striatal networks. Yamashita et al. (2006), using electron microscopy, showed that newly generated neurons form synapses with neighboring cells in the post-ischemic striatum. Additionally, a retrograde tracing study by Sun et al. (2012) showed that a subpopulation of new neurons in the damaged striatum re-establishes long connections with the substantia nigra. Moreover, Hou et al. (2008) provided both structural and electrophysiological evidence of synaptic integration of new striatal neurons after MCAO. Strong evidence in support of the regenerative role of lesion-induced neurogenesis comes from the work by Jin et al. (2010). The authors used transgenic animals expressing herpes simplex virus thymidine kinase under the control of the promoter for DCX to selectively ablate new neurons in the brain through a ganciclovir injection. They showed that neurogenesis ablation worsens the outcome after MCAO at both histological and behavioral levels.

Despite these optimistic results, the majority of these studies showed two important limitations in the restoration of neuronal circuits by adding newly generated neurons: the low survival rate of these neurons and the poor scope of their differentiation potential. Most new neurons within the ischemic striatum die, possible due to unfavorable environment in the damaged tissue (Arvidsson et al., 2002; Parent et al., 2002; Thored et al., 2006). Since generation and migration of new neurons can last for months (Thored et al., 2006; Leker et al., 2007) and even a year (Kokaia et al., 2006; Osman et al., 2011) after injury, we can assume that mature neurons may, at least partially, compensate for their high mortality within the ischemic tissue.

Another important limitation is a relevance of functional features of integrated new neurons to the pre-existing functionality of the network. Studies on a phenotype of newly generated neurons within the ischemic striatum provided contradictory results regarding a percentage of newly generated neurons expressing DARPP-32, a marker of medium-sized spiny neurons – the major class of striatal neurons. Arvidsson et al. (2002) and Parent et al. (2002) showed that after MCAO a substantial number of new striatal neurons are DARPP-32-positive. In contrast, on the same model of ischemia, Teramoto et al. (2003) and Liu et al. (2009) failed to find any newly generated DARPP-32-positive neurons at all. Teramoto et al. (2003) found that about 65% of newly generated striatal neurons are parvalbumin-positive interneurons, which normally comprise a minor subpopulation of striatal neurons. Liu et al. (2009) reported that nearly all new neurons in the damaged striatum are calretinin-positive interneurons, which are extremely rare in the striatum under physiological conditions. In addition, these new striatal neurons express the transcription factor Sp8, which is expressed in most newly generated neurons in the OB under physiological conditions. The results of Liu et al. (2009) suggested that SVZ-derived progenitors had strictly limited differentiation potency, and that after MCAO they cannot generate the majority of neuronal classes, which are lost within the damaged striatum. This point of view may be further strengthened by the work of Merkle et al. (2007). The authors found that, despite the diversity of the newly generated OB interneurons, their stem progenitors within the SVZ have a strictly restricted potency of differentiation. In fact, the SVZ stem cell niche has a mosaic organization, where NSCs from SVZ subregions vary in their differentiation potential. NSCs from a particular SVZ-subregion may give rise only to a particular type of interneurons, even when cultivated *in vitro*. It seems unlikely that these strictly predetermined cells can produce other types of neurons necessary for brain recovery.

Identification of the causes of the above-mentioned discrepancies is one of the directions for future studies. Thus, more research needs to be done to comprehensively evaluate the regenerative potential of adult neurogenesis and to develop optimal strategies for its enhancement.

## Conclusion

Up to the present time, a substantial amount of research material concerning the origins and migratory pathways of NPCs in the brain under physiological and pathological conditions has been accumulated. In the healthy brain, the majority of NPCs originate in the V-SVZ located in the walls of the lateral ventricles (Lim and Alvarez-Buylla, 2016). In the embryonic and early postnatal brain, NPCs migrate from the V-SVZ to the OB, striatum, and cortex. In adulthood, these pathways – except for migration to the OB in rodents (Lim and Alvarez-Buylla, 2016) and, probably, to the striatum in humans (Ernst et al., 2014) – remain substantially quiescent but can intensify in conditions of brain injury (Nakatomi et al., 2002; Yamashita et al., 2006; Faiz et al., 2015). Among other regions, at the very least, the adult striatum maintains the capacity to generate new neurons in both physiological (Dayer et al., 2005; Luzzati et al., 2006, 2011) and pathological (Magnusson et al., 2014; Nato et al., 2015) conditions. The fate and functionality of local newborn striatal neurons have been intensively studied (Lindvall and Kokaia, 2015; Marlier et al., 2015). Adult neurogenesis in the cortex and migration of NPCs from the V-SVZ to this area in physiological conditions are less evident (Gould et al., 2002; Dayer et al., 2005; Bhardwaj et al., 2006; Cameron and Dayer, 2008). However, whilst brain injury provokes the generation of new cortical neurons, the survival and functionality of these neurons are commonly questioned (Ohira et al., 2010; Faiz et al., 2015).

Human neurogenesis, which has been also shown in the studies with the comprehensive ^14^C-content assessment (Spalding et al., 2013), substantially differs from neurogenesis in rodents. Using this technique in studies on rodents, which are more extensively used in research, could help clarify these differences.

Despite the impressive array of research works, at present there is no full picture of neurogenesis and NPC migration in the brain. Most research works address either a single neurogenic region or a single migratory pathway of neuroblasts. Future studies on the injured brain could examine the following aspects: firstly, the local or migratory nature of NPCs after brain injury could be confirmed by novel techniques of real-time observations; secondly, since the presence of newly generated neurons does not ensure their functionality, the question about the integration of new neurons into the existing networks and their phenotypes could be further investigated; thirdly, comparative studies are needed to understand the difference between human and rodent neurogenesis for future translation of new therapies to clinic.

It is likely that in the future, methods for longitudinal observation capable of capturing new neuron production, migration, and functional integration in the same animal brain, will be improved. We believe that the advancement of methods for *in vivo* visualization of neurogenesis in the brain could fundamentally change the current situation in this field.

## Author Contributions

NN-D wrote the manuscript. MK conceived the presented idea and critically revised the manuscript. Both authors contributed to the literature search and analyses.

## Conflict of Interest Statement

The authors declare that the research was conducted in the absence of any commercial or financial relationships that could be construed as a potential conflict of interest.

## Abbreviations

V-SVZ: ventricular-subventricular zone

## References

[B1] Abdipranoto-Cowley A. Park J. S. Croucher D. Daniel J. Henshall S. Galbraith S. (2009). Activin a is essential for neurogenesis following neurodegeneration. *Stem Cells* 27 1330–1346. 10.1002/stem.80 19489097 PMC2733378

[B2] Abrous D. N. Koehl M. Le Moal M. (2005). Adult neurogenesis: from precursors to network and physiology. *Physiol. Rev.* 85 523–569. 10.1152/physrev.00055.2003 15788705

[B3] Ahmed A. I. Shtaya A. B. Zaben M. J. Owens E. V. Kiecker C. Gray W. P. (2012). Endogenous GFAP-positive neural stem/progenitor cells in the postnatal mouse cortex are activated following traumatic brain injury. *J. Neurotrauma* 29 828–842. 10.1089/neu.2011.1923 21895532 PMC3303093

[B4] Ajioka I. Jinnou H. Okada K. Sawada M. Saitoh S. Sawamoto K. (2015). Enhancement of neuroblast migration into the injured cerebral cortex using laminin-containing porous sponge. *Tissue Eng. Part A* 21 193–201. 10.1089/ten.tea.2014.0080 25010638

[B5] Altman J. (1962). Are new neurons formed in the brains of adult mammals? *Science* 135 1127–1128. 13860748 10.1126/science.135.3509.1127

[B6] Alvarez-Buylla A. García-Verdugo J. M. Tramontin A. D. (2001). A unified hypothesis on the lineage of neural stem cells. *Nat. Rev. Neurosci.* 2 287–293. 10.1038/35067582 11283751

[B7] Alvarez-Buylla A. Seri B. Doetsch F. (2002). Identification of neural stem cells in the adult vertebrate brain. *Brain Res. Bull.* 57 751–758. 10.1016/S0361-9230(01)00770-5 12031271

[B8] Arvidsson A. Collin T. Kirik D. Kokaia Z. Lindvall O. (2002). Neuronal replacement from endogenous precursors in the adult brain after stroke. *Nat. Med.* 9 548–553. 10.1038/nm747 12161747

[B9] Aschauer D. F. Kreuz S. Rumpel S. (2013). Analysis of transduction efficiency, tropism and axonal transport of AAV serotypes 1, 2, 5, 6, 8 and 9 in the mouse brain. *PLoS One* 8:e076310. 10.1371/journal.pone.0076310 24086725 PMC3785459

[B10] Bendel O. Bueters T. von Euler M. Ove Ogren S. Sandin J. von Euler G. (2005). Reappearance of Hippocampal CA1 neurons after ischemia is associated with recovery of learning and memory. *J. Cereb. Blood Flow Metab.* 25 1586–1595. 10.1038/sj.jcbfm.9600153 15917746

[B11] Bergami M. Masserdotti G. Temprana S. G. Motori E. Eriksson T. M. Göbel J. (2015). A critical period for experience-dependent remodeling of adult-born neuron connectivity. *Neuron* 85 710–717. 10.1016/j.neuron.2015.01.001 25661179

[B12] Bernier P. J. Vinet J. Cossette M. Parent A. (2000). Characterization of the subventricular zone of the adult human brain: evidence for the involvement of Bcl-2. *Neurosci. Res.* 37 67–78. 10802345 10.1016/s0168-0102(00)00102-4

[B13] Bhardwaj R. D. Curtis M. A. Spalding K. L. Buchholz B. A. Fink D. Bjork-Eriksson T. (2006). Neocortical neurogenesis in humans is restricted to development. *Proc. Natl. Acad. Sci.* 103 12564–12568. 10.1073/pnas.0605177103 16901981 PMC1567918

[B14] Bi B. Salmaso N. Komitova M. Simonini M. V. Silbereis J. Cheng E. (2011). Cortical GFAP positive cells generate neurons after perinatal hypoxic injury. *J. Neurosci.* 31 9205–9221. 10.1523/JNEUROSCI.0518-11.2011 21697371 PMC3142780

[B15] Bonaguidi M. A. Wheeler M. A. Shapiro J. S. Stadel R. P. Sun G. J. Ming G. L. (2011). In vivo clonal analysis reveals self-renewing and multipotent adult neural stem cell characteristics. *Cell* 145 1142–1155. 10.1016/j.cell.2011.05.024 21664664 PMC3124562

[B16] Buffo A. Rite I. Tripathi P. Lepier A. Colak D. Horn A. P. (2008). Origin and progeny of reactive gliosis: a source of multipotent cells in the injured brain. *Proc. Natl. Acad. Sci. U.S.A.* 105 3581–3586. 10.1073/pnas.0709002105 18299565 PMC2265175

[B17] Bull N. D. Bartlett P. F. (2005). The adult mouse hippocampal progenitor is neurogenic but not a stem cell. *J. Neurosci.* 25 10815–10821. 10.1523/JNEUROSCI.3249-05.2005 16306394 PMC6725873

[B18] Bulte J. W. Brooks R. A. Moskowitz B. M. Bryant L. H. Frank J. A. (1999). Relaxometry and magnetometry of the MR contrast agent MION-46L. *Mag. Res. Med.* 42 379–384. 10440963 10.1002/(sici)1522-2594(199908)42:2<379::aid-mrm20>3.0.co;2-l

[B19] Cameron H. A. Dayer A. G. (2008). New interneurons in the adult neocortex: small, sparse, but significant? *Biol. Psychiatry* 63 650–655. 18067877 10.1016/j.biopsych.2007.09.023 PMC2423203

[B20] Chen J. Magavi S. S. Macklis J. D. (2004). Neurogenesis of corticospinal motor neurons extending spinal projections in adult mice. *Proc. Natl. Acad. Sci.* 101 16357–16362. 10.1073/pnas.0406795101 15534207 PMC528970

[B21] Cheyuo C. Aziz M. Yang W.-L. Jacob A. Zhou M. Wang P. (2015). Milk fat globule-EGF factor VIII attenuates CNS injury by promoting neural stem cell proliferation and migration after cerebral ischemia. *PLoS One* 10:e0122833. 10.1371/journal.pone.0122833 25867181 PMC4394995

[B22] Chung K. Deisseroth K. (2013). CLARITY for mapping the nervous system. *Nat. Methods* 10 508–513. 10.1038/nmeth.2481 23722210

[B23] Covey M. V. Jiang Y. Alli V. V. Yang Z. Levison S. W. (2010). “Defining the critical period for neocortical neurogenesis after pediatric brain injury”. *Dev. Neurosci.* 32 488–498. 10.1159/000321607 21160158

[B24] Cui X. Chen J. Zacharek A. Roberts C. Yang Y. Chopp M. (2009). Nitric oxide donor up-regulation of SDF1/CXCR4 and Ang1/Tie2 promotes neuroblast cell migration after stroke. *J. Neurosci. Res.* 87 86–95. 10.1002/jnr.21836 18711749 PMC2606920

[B25] Daval J. L. Pourié G. Grojean S. Lièvre V. Strazielle C. Blaise S. (2004). Neonatal hypoxia triggers transient apoptosis followed by neurogenesis in the rat CA1 hippocampus. *Pediatr. Res.* 55 561–567. 10.1203/01.PDR.0000113771.51317.37 14739363

[B26] Davidsson M. Diaz-Fernandez P. Schwich O. D. Torroba M. Wang G. Björklund T. (2016). A novel process of viral vector barcoding and library preparation enables high-diversity library generation and recombination-free paired-end sequencing. *Sci. Rep.* 6:37563. 10.1038/srep37563 27874090 PMC5118689

[B27] Dayer A. G. Cleaver K. M. Abouantoun T. Cameron H. A. (2005). New GABAergic interneurons in the adult neocortex and striatum are generated from different precursors. *J. Cell Biol.* 168 415–427. 10.1083/jcb.200407053 15684031 PMC2171716

[B28] Dayer A. G. Jenny B. Potter G. Sauvain M. O. Szabó G. Vutskits L. (2008). Recruiting new neurons from the subventricular zone to the rat postnatal cortex: an organotypic slice culture model. *Eur. J. Neurosci.* 27 1051–1060. 10.1111/j.1460-9568.2008.06091.x 18364030

[B29] de Jong N. W. M. van der Horst T. van Strijp J. A. G. Nijland R. (2017). Fluorescent reporters for markerless genomic integration in *Staphylococcus aureus*. *Sci. Rep.* 7:43889. 10.1038/srep43889 28266573 PMC5339689

[B30] De Marchis S. Fasolo A. Puche A. C. (2004). Subventricular zone-derived neuronal progenitors migrate into the subcortical forebrain to postnatal mice. *J. Comp. Neurol.* 476 290–300. 10.1002/cne.20217 15269971

[B31] Denk W. Strickler J. H. Webb W. W. (1990). Two-photon laser scanning fluorescence microscopy. *Science* 245 843–845. 10.1126/science.245.4920.843 2321027

[B32] Dennis C. V. Suh L. S. Rodriguez M. L. Kril J. J. Sutherland G. T. (2016). Human adult neurogenesis across the ages: an immunohistochemical study. *Neuropathol. Appl. Neurobiol.* 42 621–638. 10.1111/nan.12337 27424496 PMC5125837

[B33] Doetsch F. (2003). The glial identity of neural stem cells. *Nat. Neurosci.* 6 1127–1134. 10.1038/nn1144 14583753

[B34] Doetsch F. Caillé I. Lim D. A. García-Verdugo J. M. Alvarez-Buylla A. (1999). Subventricular zone astrocytes are neural stem cells in the adult mammalian brain. *Cell* 97 703–716. 10.1016/S0092-8674(00)80783-7 10380923

[B35] Doetsch F. García-Verdugo J. M. Alvarez-Buylla A. (1997). Cellular composition and three-dimensional organization of the subventricular germinal zone in the adult mammalian brain. *J. Neurosci.* 17 5046–5061. 10.1523/JNEUROSCI.17-13-05046.1997 9185542 PMC6573289

[B36] Duan C. L. Liu C. W. Shen S. W. Yu Z. Mo J. L. Chen X. H. (2015). Striatal astrocytes transdifferentiate into functional mature neurons following ischemic brain injury. *Glia* 63 1660–1670. 10.1002/glia.22837 26031629 PMC5033006

[B37] Duan X. Kang E. Liu C. Y. Ming G. L. Song H. (2008). Development of neural stem cell in the adult brain. *Curr. Opin. Neurobiol.* 18 108–115. 10.1016/j.conb.2008.04.001 18514504 PMC2464621

[B38] Ehninger D. Kempermann G. (2003). Regional effects of wheel running and environmental enrichment on cell genesis and microglia proliferation in the adult murine neocortex. *Cereb. Cortex* 13 845–851. 10.1093/cercor/13.8.845 12853371

[B39] Elvira G. García I. Benito M. Gallo J. Desco M. Penadés S. (2012). Live imaging of mouse endogenous neural progenitors migrating in response to an induced tumor. *PLoS One* 7:e44466. 10.1371/journal.pone.0044466 22957072 PMC3434138

[B40] Encinas J. M. Michurina T. V. Peunova N. Park J. H. Tordo J. Peterson D. A. (2011). Division-coupled astrocytic differentiation and age-related depletion of neural stem cells in the adult hippocampus. *Cell Stem Cell* 8 566–579. 10.1016/j.stem.2011.03.010 21549330 PMC3286186

[B41] Eriksson P. S. Perfilieva E. Björk-Eriksson T. Alborn A. M. Nordborg C. Peterson D. A. (1998). Neurogenesis in the adult human hippocampus. *Nat. Med.* 4 1313–1317. 10.1038/3305 9809557

[B42] Ernst A. Alkass K. Bernard S. Salehpour M. Perl S. Tisdale J. (2014). Neurogenesis in the striatum of the adult human brain. *Cell* 156 1072–1083. 10.1016/j.cell.2014.01.044 24561062

[B43] Faiz M. Acarin L. Villapol S. Schulz S. Castellano B. Gonzalez B. (2008). Substantial migration of SVZ cells to the cortex results in the generation of new neurons in the excitotoxically damaged immature rat brain. *Mol. Cell. Neurosci.* 38 170–182. 10.1016/j.mcn.2008.02.002 18434192

[B44] Faiz M. Sachewsky N. Gascón S. Bang K. W. Morshead C. M. Nagy A. (2015). Adult neural stem cells from the subventricular zone give rise to reactive astrocytes in the cortex after stroke. *Cell Stem Cell* 17 624–634. 10.1016/j.stem.2015.08.002 26456685

[B45] Fuentealba L. C. Rompani S. B. Parraguez J. I. Obernier K. Romero R. Cepko C. L. (2015). Embryonic origin of postnatal neural stem cells. *Cell* 161 1644–1655. 10.1016/j.cell.2015.05.041 26091041 PMC4475276

[B46] Fujioka T. Kaneko N. Ajioka I. Nakaguchi K. Omata T. Ohba H. (2017). B1 integrin signaling promotes neuronal migration along vascular scaffolds in the post-stroke brain. *EBioMedicine* 16 195–203. 10.1016/j.ebiom.2017.01.005 28153772 PMC5474439

[B47] Fukuzaki Y. Shin H. Kawai H. D. Yamanoha B. Kogure S. (2015). 532 Nm low-power laser irradiation facilitates the migration of GABAergic neural stem/progenitor cells in mouse neocortex. *PLoS One* 10:e0123833. 10.1371/journal.pone.0123833 25919297 PMC4412395

[B48] Gage F. H. (2000). Mammalian neural stem cells. *Science* 287 1433–1438. 10.1126/science.287.5457.1433 10688783

[B49] Goings G. E. Sahni V. Szele F. G. (2004). Migration patterns of subventricular zone cells in adult mice change after cerebral cortex injury. *Brain Res.* 996 213–226. 10.1016/j.brainres.2003.10.034 14697499

[B50] Goldman S. A. Nottebohm F. (2006). Neuronal production, migration, and differentiation in a vocal control nucleus of the adult female canary brain. *Proc. Natl. Acad. Sci.* 80 2390–2394. 10.1073/pnas.80.8.2390 6572982 PMC393826

[B51] Gomez-Nicola D. Riecken K. Fehse B. Perry V. H. (2014). In-Vivo RGB marking and multicolour single-cell tracking in the adult brain. *Sci. Rep.* 4:7520. 10.1038/srep07520 25531807 PMC4273606

[B52] Gonçalves J. T. Schafer S. T. Gage F. H. (2016). Adult neurogenesis in the hippocampus: from stem cells to behavior. *Cell* 167 897–914. 10.1016/j.cell.2016.10.021 27814520

[B53] Gotts J. E. Chesselet M. F. (2005). Migration and fate of newly born cells after focal cortical ischemia in adult rats. *J. Neurosci. Res.* 80 160–171. 10.1002/jnr.20434 15751027

[B54] Gould E. Vail N. Wagers M. Gross C. G. (2002). Adult-generated hippocampal and neocortical neurons in macaques have a transient existence. *Proc. Natl. Acad. Sci. U.S.A.* 98 10910–10917. 10.1073/pnas.181354698 11526209 PMC58573

[B55] Grade S. Weng Y. C. Snapyan M. Kriz J. Malva J. O. Saghatelyan A. (2013). Brain-derived neurotrophic factor promotes vasculature-associated migration of neuronal precursors toward the ischemic striatum. *PLoS One* 8:e55039. 10.1371/journal.pone.0055039 23383048 PMC3558494

[B56] Gu W. Brännström T. Wester P. (2000). Cortical neurogenesis in adult rats after reversible photothrombotic stroke. *J. Cereb. Blood Flow Metab.* 20 1166–1173. 10.1097/00004647-200008000-00002 10950377

[B57] Hou S. W. Wang Y. Q. Xu M. Shen D. H. Wang J. J. Huang F. (2008). Functional integration of newly generated neurons into striatum after cerebral ischemia in the adult rat brain. *Stroke* 39 2837–2844. 10.1161/STROKEAHA.107.510982 18635857

[B58] Ihunwo A. O. Tembo L. H. Dzamalala C. (2016). The dynamics of adult neurogenesis in human hippocampus. *Neural Regen. Res.* 11 1869–1883. 10.4103/1673-5374.195278 28197172 PMC5270414

[B59] Imayoshi I. Sakamoto M. Ohtsuka T. Takao K. Miyakawa T. Yamaguchi M. (2008). Roles of continuous neurogenesis in the structural and functional integrity of the adult forebrain. *Nat. Neurosci.* 11 1153–1161. 10.1038/nn.2185 18758458

[B60] Inta D. Alfonso J. von Engelhardt J. Kreuzberg M. M. Meyer A. H. van Hooft J. A. (2008). Neurogenesis and widespread forebrain migration of distinct GABAergic neurons from the postnatal subventricular zone. *Proc. Natl. Acad. Sci.* 105 20994–20999. 10.1073/pnas.0807059105 19095802 PMC2605417

[B61] Iordanova B. Ahrens E. T. (2012). In vivo magnetic resonance imaging of ferritin-based reporter visualizes native neuroblast migration. *NeuroImage* 59 1004–1012. 10.1016/j.neuroimage.2011.08.068 21939774 PMC3230706

[B62] Jensen K. H. R. Berg R. W. (2017). Advances and perspectives in tissue clearing using CLARITY. *J. Chem. Neuroanat.* 86 19–34. 10.1016/j.jchemneu.2017.07.005 28728966

[B63] Jessberger S. Gage F. H. (2014). Adult neurogenesis: bridging the gap between mice and humans. *Trends Cell Biol.* 24 558–563. 10.1016/j.tcb.2014.07.003 25124338

[B64] Jin K. Sun Y. Xie L. Peel A. Mao X. O. Batteur S. (2003). Directed migration of neuronal precursors into the ischemic cerebral cortex and striatum. *Mol. Cell. Neurosci.* 24 171–189. 10.1016/S1044-7431(03)00159-3 14550778

[B65] Jin K. Wang X. Xie L. Mao X. O. Greenberg D. A. (2010). Transgenic ablation of doublecortin-expressing cells suppresses adult neurogenesis and worsens stroke outcome in mice. *Proc. Natl. Acad. Sci. U.S.A.* 107 7993–7998. 10.1073/pnas.1000154107 20385829 PMC2867852

[B66] Kaplan M. S. Hinds J. W. (1977). “Neurogenesis in the adult rat: electron microscopic analysis of light radioautographs”. *Science* 197 1092–1094. 887941 10.1126/science.887941

[B67] Kazanis I. Gorenkova N. Zhao J. W. Franklin R. J. Modo M. Ffrench-Constant C. (2013). The late response of rat subependymal zone stem and progenitor cells to stroke is restricted to directly affected areas of their niche. *Exp. Neurol.* 248 387–397. 10.1016/j.expneurol.2013.06.025 23830949 PMC3782662

[B68] Kempermann G. Gage F. H. Aigner L. Song H. Curtis M. A. Thuret S. (2018). Human adult neurogenesis: evidence and remaining questions. *Cell Stem Cell* 23 25–30. 10.1016/j.stem.2018.04.004 29681514 PMC6035081

[B69] Khodanovich M. Y. Kisel A. A. Chernysheva G. A. Smolyakova V. I. Savchenko R. R. Plotnikov M. B. (2016). Effect of fluoxetine on neurogenesis in hippocampal dentate gyrus after global transient cerebral ischemia in rats. *Bull. Exp. Biol. Med.* 161 351–354. 10.1007/s10517-016-3412-4 27496030

[B70] Khodanovich M. Y. Kisel A. A. Kudabaeva M. S. Chernysheva G. A. Smolyakova V. I. Glazacheva V. Y. (2018a). Abnormal migration of immature neurons in the global cerebral ischemia model. *Am. Neurol. Assoc. Ann. Meet.* 84 154–155. 10.1002/ana.25331

[B71] Khodanovich M. Y. Kisel A. A. Kudabaeva M. S. Chernysheva G. A. Smolyakova V. I. Krutenkova E. P. (2018b). Effects of fluoxetine on hippocampal neurogenesis and neuroprotection in the model of global cerebral ischemia in rats. *Int. J. Mol. Sci.* 19 12–24. 10.3390/ijms19010162 29304004 PMC5796111

[B72] Kobayashi T. Ahlenius H. Thored P. Kobayashi R. Kokaia Z. Lindvall O. (2006). Intracerebral infusion of glial cell line-derived neurotrophic factor promotes striatal neurogenesis after stroke in adult rats. *Stroke* 37 2361–2367. 10.1161/01.STR.0000236025.44089.e1 16873711

[B73] Kojima T. Hirota Y. Ema M. Takahashi S. Miyoshi I. Okano H. (2010). Subventricular zone-derived neural progenitor cells migrate along a blood vessel scaffold toward the post-stroke striatum. *Stem Cells* 28 545–554. 10.1002/stem.306 20073084

[B74] Kokaia Z. Thored P. Arvidsson A. Lindvall O. (2006). Regulation of stroke-induced neurogenesis in adult brain-recent scientific progress. *Cereb. Cortex* 16(Suppl. 1), i162–i167. 10.1093/cercor/bhj174 16766702

[B75] Kolb B. Morshead C. Gonzalez C. Kim M. Gregg C. Shingo T. (2007). Growth factor-stimulated generation of new cortical tissue and functional recovery after stroke damage to the motor cortex of rats. *J. Cereb. Blood Flow Metab.* 27 983–997. 10.1038/sj.jcbfm.9600402 16985505

[B76] Kornack D. R. Rakic P. (2001). The generation, migration, and differentiation of olfactory neurons in the adult primate brain. *Proc. Natl. Acad. Sci. U.S.A.* 98 4752–4757. 10.1073/pnas.081074998 11296302 PMC31906

[B77] Kreuzberg M. Kanov E. Timofeev O. Schwaninger M. Monyer H. Khodosevich K. (2010). Increased subventricular zone-derived cortical neurogenesis after ischemic lesion. *Exp. Neurol.* 226 90–99. 10.1016/j.expneurol.2010.08.006 20713052

[B78] Kuge A. Takemura S. Kokubo Y. Sato S. Goto K. Kayama T. (2009). Temporal profile of neurogenesis in the subventricular zone, dentate gyrus and cerebral cortex following transient focal cerebral ischemia. *Neurol. Res.* 31 969–976. 10.1179/174313209X383312 19138475

[B79] Kuhn H. G. Eisch A. J. Spalding K. Peterson D. A. (2016). Detection and phenotypic characterization of adult neurogenesis. *Cold Spring Harb. Perspect. Biol.* 8:a025981. 10.1101/cshperspect.a025981 26931327 PMC4772100

[B80] Lai B. Mao X. O. Xie L. Jin K. Greenberg D. A. (2008). Electrophysiological neurodifferentiation of subventricular zonederived precursor cells following stroke. *Neurosci. Lett.* 442 305–308. 10.1016/j.neulet.2008.07.032 18647640 PMC2547133

[B81] Landecker H. (2009). Seeing things: from microcinematography to live cell imaging. *Nat. Methods* 6 707–709. 10.1038/nmeth1009-707 19953685

[B82] Laywell E. D. Rakic P. Kukekov V. G. Holland E. C. Steindler D. A. (2000). Identification of a multipotent astrocytic stem cell in the immature and adult mouse brain. *Proc. Natl. Acad. Sci. U.S.A.* 97 13883–13888. 10.1073/pnas.250471697 11095732 PMC17670

[B83] Le Magueresse C. Alfonso J. Bark C. Eliava M. Khrulev S. Monyer H. (2012). Subventricular zone-derived neuroblasts use vasculature as a scaffold to migrate radially to the cortex in neonatal mice. *Cereb. Cortex* 22 2285–2296. 10.1093/cercor/bhr302 22095212

[B84] Lee S. R. Kim H. Y. Rogowska J. Zhao B. Q. Bhide P. Parent J. M. (2006). Involvement of matrix metalloproteinase in neuroblast cell migration from the subventricular zone after stroke. *J. Neurosci.* 26 3491–3495. 10.1523/JNEUROSCI.4085-05.2006 16571756 PMC6673870

[B85] Leker R. R. Soldner F. Velasco I. Gavin D. K. Androutsellis-Theotokis A. McKay R. D. (2007). Long-lasting regeneration after ischemia in the cerebral cortex. *Stroke* 38 153–161. 10.1161/01.STR.0000252156.65953.a9 17122419

[B86] Li B. Piao C. S. Liu X. Y. Guo W. P. Xue Y. Q. Duan W. M. (2010). Brain self-protection: the role of endogenous neural progenitor cells in adult brain after cerebral cortical ischemia. *Brain Res.* 1327 91–102. 10.1016/j.brainres.2010.02.030 20171958

[B87] Li G. Fang L. Fernández G. Pleasure S. J. (2013). The ventral hippocampus is the embryonic origin for adult neural stem cells in the dentate gyrus. *Neuron* 78 658–672. 10.1016/j.neuron.2013.03.019 23643936 PMC3669230

[B88] Li L. Harms K. M. Ventura P. B. Lagace D. C. Eisch A. J. Cunningham L. A. (2010). Focal cerebral ischemia induces a multilineage cytogenic response from adult subventricular zone that is predominantly gliogenic. *Magn. Reson. Imaging* 58 1610–1619. 10.1016/j.immuni.2010.12.017.Two-stage 20578055 PMC2919586

[B89] Li W. L. Yu S. P. Ogle M. E. Ding X. S. Wei L. (2008). Enhanced neurogenesis and cell migration following focal ischemia and peripheral stimulation in mice. *Dev. Neurobiol.* 68 1474–1486. 10.1002/dneu.20674 18777565 PMC2756802

[B90] Li Y. Yu S. P. Mohamad O. Genetta T. Wei L. (2011). Sublethal transient global ischemia stimulates migration of neuroblasts and neurogenesis in mice. *Stroke* 1 184–196. 10.1007/s12975-010-0016-6.Sublethal 21792374 PMC3142584

[B91] Lim D. A. Alvarez-Buylla A. (2016). The adult ventricular – subventricular zone and olfactory bulb neurogenesis. *Cold Spring Harb. Perspect. Biol.* 8:a018820. 10.1101/cshperspect.a018820 27048191 PMC4852803

[B92] Lindvall O. Kokaia Z. (2015). Neurogenesis following stroke affecting the adult brain. *Cold Spring Harb. Perspect. Biol.* 7 1–20. 10.1101/cshperspect.a019034 26525150 PMC4632663

[B93] Liu F. You Y. Li X. Ma T. Nie Y. Wei B. (2009). Brain injury does not alter the intrinsic differentiation potential of adult neuroblasts. *J. Neurosci.* 29 5075–5087. 10.1523/JNEUROSCI.0201-09.2009 19386903 PMC6665479

[B94] Liu S. Trapnell C. (2016). Single-cell transcriptome sequencing: recent advances and remaining challenges. *F1000Res.* 5:182. 10.12688/f1000research.7223.1 26949524 PMC4758375

[B95] Livet J. Weissman T. A. Kang H. Draft R. W. Lu J. Bennis R. A. (2007). Transgenic strategies for combinatorial expression of fluorescent proteins in the nervous system. *Nature* 450 56–62. 10.1038/nature06293 17972876

[B96] Lledo P. M. Saghatelyan A. (2005). Integrating new neurons into the adult olfactory bulb: joining the network, life-death decisions, and the effects of sensory experience. *Trends Neurosci.* 28 248–254. 10.1016/j.tins.2005.03.005 15866199

[B97] Llorens-Bobadilla E. Zhao S. Baser A. Saiz-Castro G. Zwadlo K. Martin-Villalba A. (2015). Single-cell transcriptomics reveals a population of dormant neural stem cells that become activated upon brain injury. *Cell Stem Cell* 17 329–340. 10.1016/j.stem.2015.07.002 26235341

[B98] Lugert S. Vogt M. Tchorz J. S. Müller M. Giachino C. Taylor V. (2012). Homeostatic neurogenesis in the adult hippocampus does not involve amplification of ascl1 high intermediate progenitors. *Nat. Commun.* 14:670. 10.1038/ncomms1670 22334073

[B99] Luzzati F. De Marchis S. Fasolo A. Peretto P. (2006). Neurogenesis in the caudate nucleus of the adult rabbit. *J. Neurosci.* 26 609–621. 10.1523/jneurosci.4371-05.2006 16407559 PMC6674396

[B100] Luzzati F. De Marchis S. Parlato R. Gribaudo S. Schütz G. Fasolo A. (2011). New striatal neurons in a mouse model of progressive striatal degeneration are generated in both the subventricular zone and the striatal parenchyma. *PLoS One* 6:e025088. 10.1371/journal.pone.0025088 21980380 PMC3184103

[B101] Luzzati F. Nato G. Oboti L. Vigna E. Rolando C. Armentano M. (2014). Quiescent neuronal progenitors are activated in the juvenile guinea pig lateral striatum and give rise to transient neurons. *Development* 141 4065–4075. 10.1242/dev.107987 25336736

[B102] Madsen T. M. Yeh D. D. Valentine G. W. Duman R. S. (2005). Electroconvulsive seizure treatment increases cell proliferation in rat frontal cortex. *Neuropsychopharmacology* 30 27–34. 10.1038/sj.npp.1300565 15383831

[B103] Magavi S. S. Leavitt B. R. Macklis J. D. (2000). Induction of neurogenesis in the neocortex of adult mice. *Nature* 405 951–955. 10.1038/35016083 10879536

[B104] Magnusson J. P. Göritz C. Tatarishvili J. Dias D. O. Smith E. M. Lindvall O. (2014). A latent neurogenic program in astrocytes regulated by notch signaling in the mouse. *Science* 346 237–241. 10.1126/science.346.6206.237 25301628

[B105] Marlier Q. Verteneuil S. Vandenbosch R. Malgrange B. (2015). Mechanisms and functional significance of stroke-induced neurogenesis. *Front. Neurosci.* 8:458 10.3389/fnins.2015.00458 PMC4672088 26696816

[B106] Marques B. L. Carvalho G. A. Freitas E. M. M. Chiareli R. A. Barbosa T. G. Di Araújo A. G. P. (2019). The role of neurogenesis in neurorepair after ischemic stroke. *Semin. Cell Dev. Biol.* 10.1016/j.semcdb.2018.12.003 [Epub ahead of print]. 30550812

[B107] Masuda T. Isobe Y. Aihara N. Furuyama F. Misumi S. Kim T. S. (2007). Increase in neurogenesis and neuroblast migration after a small intracerebral hemorrhage in rats. *Neurosci. Lett.* 425 114–119. 10.1016/j.neulet.2007.08.039 17826909

[B108] Merkle F. T. Mirzadeh Z. Alvarez-Buylla A. (2007). Mosaic organization of neural stem cells in the adult brain. *Science* 317 381–384. 10.1126/science.1144914 17615304

[B109] Mignone J. Peunova N. Enikolopov G. (2016). “Nestin-Based Reporter Transgenic Mouse Lines,” in *Multipotent Stem Cells of the Hair Follicle. Methods in Molecular Biology* Vol. 1453 ed. Hoffman R. (New York, NY: Humana Press). 10.1007/978-1-4939-3786-8_2 27431241

[B110] Ming G. L. Song H. (2005). Adult neurogenesis in the mammalian central nervous system. *Ann. Rev. Neurosci.* 28 223–250. 10.1146/annurev.neuro.28.051804.101459 16022595

[B111] Ming G. L. Song H. (2011). Adult neurogenesis in the mammalian brain: significant answers and significant questions. *Neuron* 70 687–702. 10.1016/j.neuron.2011.05.001 21609825 PMC3106107

[B112] Mizrak D. Levitin H. M. Delgado A. C. Crotet V. Yuan J. Chaker Z. (2019). Single-cell analysis of regional differences in adult V-SVZ neural stem cell lineages. *Cell Rep.* 26 394.e5–406.e5. 10.1016/j.celrep.2018.12.044 30625322 PMC6368857

[B113] Moraga A. Pradillo J. M. Cuartero M. I. Hernández-Jiménez M. Oses M. Moro M. A. (2014). Toll-like receptor 4 modulates cell migration and cortical neurogenesis after focal cerebral ischemia. *FASEB J.* 28 4710–4718. 10.1096/fj.14-252452 25063846

[B114] Moreno-Jiménez E. P. Flor-García M. Terreros-Roncal J. Rábano A. Cafini F. Pallas-Bazarra N. (2019). Adult hippocampal neurogenesis is abundant in neurologically healthy subjects and drops sharply in patients with alzheimer’s disease. *Nat. Med.* 25 554–560. 10.1038/s41591-019-0375-9 30911133

[B115] Müller-Taubenberger A. Ishikawa-Ankerhold H. C. (2014). Fluorescent reporters and methods to analyze fluorescent signals. *Eur. J. Histochem.* 57:9 10.4081/ejh.2013.br9 23494303

[B116] Nacher J. Bonfanti L. (2015). New neurons from old beliefs in the adult piriform cortex? a commentary on: ‘occurrence of new neurons in the piriform cortex’. *Front. Neuroanat.* 9:62 10.3389/fnana.2015.00062 PMC4440910 26052272

[B117] Nakagomi T. Kubo S. Nakano-Doi A. Sakuma R. Lu S. Narita A. (2015). Brain vascular pericytes following ischemia have multipotential stem cell activity to differentiate into neural and vascular lineage cells. *Stem Cells* 33 1962–1974. 10.1002/stem.1977 25694098

[B118] Nakagomi T. Molnár Z. Nakano-Doi A. Taguchi A. Saino O. Kubo S. (2011). Ischemia-induced neural stem/progenitor cells in the pia mater following cortical infarction. *Stem Cells Dev.* 20 2037–2051. 10.1089/scd.2011.0279 21838536

[B119] Nakaguchi K. Jinnou H. Kaneko N. Sawada M. Hikita T. Saitoh S. (2012). Growth factors released from gelatin hydrogel microspheres increase new neurons in the adult mouse brain. *Stem Cells Int.* 2012:915160. 10.1155/2012/915160 23093979 PMC3474987

[B120] Nakatomi H. Kuriu T. Okabe S. Yamamoto S. Hatano O. Kawahara N. (2002). Regeneration of hippocampal pyramidal neurons after ischemic brain injury by recruitment of endogenous neural progenitors. *Cell* 110 429–441. 12202033 10.1016/s0092-8674(02)00862-0

[B121] Nato G. Caramello A. Trova S. Avataneo V. Rolando C. Taylor V. (2015). Striatal astrocytes produce neuroblasts in an excitotoxic model of huntington’s disease. *Development* 142 840–845. 10.1242/dev.116657 25655705

[B122] Naumova A. V. Modo M. Moore A. Murry C. E. Frank J. A. (2014). Clinical imaging in regenerative medicine. *Nat. Biotechnol.* 32 804–818. 10.1038/nbt.2993 25093889 PMC4164232

[B123] Nieman B. J. Shyu J. Y. Rodriguez J. J. Garcia A. D. Joyner A. L. Turnbull D. H. (2010). In vivo MRI of neural cell migration dynamics in the mouse brain. *Neuroimage* 50 456–464. 10.1016/j.neuroimage.2009.12.107.In 20053381 PMC2899477

[B124] Niv F. Keiner S. Krishna Witte O. W. Lie D. C. Redecker C. (2012). Aberrant neurogenesis after stroke: a retroviral cell labeling study. *Stroke* 43 2468–2475. 10.1161/STROKEAHA.112.660977 22738919

[B125] Norman A. B. Thomas S. R. Pratt R. G. Lu S. Y. Norgren R. B. (1992). Magnetic resonance imaging of neural transplants in rat brain using a superparamagnetic contrast agent. *Brain Res.* 594 279–283. 10.1016/0006-8993(92)91135-2 1450953

[B126] Obernier K. Cebrian-Silla A. Thomson M. Parraguez J. I. Anderson R. Guinto C. (2018). Adult neurogenesis is sustained by symmetric self-renewal and differentiation. *Cell Stem Cell* 22 221.e8–234.e8. 10.1016/j.stem.2018.01.003 29395056 PMC5802882

[B127] Ohab J. J. Fleming S. Blesch A. Carmichael S. T. (2006). A neurovascular niche for neurogenesis after stroke. *J. Neurosci.* 26 13007–13016. 10.1523/JNEUROSCI.4323-06.2006 17167090 PMC6674957

[B128] Ohira K. Furuta T. Hioki H. Nakamura K. C. Kuramoto E. Tanaka Y. (2010). Ischemia-induced neurogenesis of neocortical layer 1 progenitor cells. *Nat. Neurosci.* 13 173–179. 10.1038/nn.2473 20037576

[B129] Ortega F. J. Jolkkonen J. Mahy N. Rodríguez M. J. (2013). Glibenclamide enhances neurogenesis and improves long-term functional recovery after transient focal cerebral ischemia. *J. Cereb. Blood Flow Metab.* 33 356–364. 10.1038/jcbfm.2012.166 23149556 PMC3587805

[B130] Osman A. M. Porritt M. J. Nilsson M. Kuhn H. G. (2011). Long-term stimulation of neural progenitor cell migration after cortical ischemia in mice. *Stroke* 42 3559–3565. 10.1161/STROKEAHA.111.627802 21980195

[B131] Oya S. Yoshikawa G. Takai K. Tanaka J. I. Higashiyama S. Saito N. (2009). Attenuation of notch signaling promotes the differentiation of neural progenitors into neurons in the hippocampal ca1 region after ischemic injury. *Neuroscience* 158 683–692. 10.1016/j.neuroscience.2008.10.043 19017538

[B132] Palma-Tortosa S. García-Culebras A. Moraga A. Hurtado O. Perez-Ruiz A. Durán-Laforet V. (2017). Specific features of SVZ neurogenesis after cortical ischemia: a longitudinal study. *Sci. Rep.* 7:16343. 10.1038/s41598-017-16109-7 29180821 PMC5703956

[B133] Panizzo R. A. Kyrtatos P. G. Price A. N. Gadian D. G. Ferretti P. Lythgoe M. F. (2009). In vivo magnetic resonance imaging of endogenous neuroblasts labelled with a ferumoxide-polycation complex. *NeuroImage* 44 1239–1246. 10.1016/j.neuroimage.2008.10.062 19059485

[B134] Parent J. M. Vexler Z. S. Gong C. Derugin N. Ferriero D. M. (2002). Rat forebrain neurogenesis and striatal neuron replacement after focal stroke. *Ann. Neurol.* 52 802–813. 10.1002/ana.10393 12447935

[B135] Pauly M. C. Piroth T. Döbrössy M. Nikkhah G. (2012). Restoration of the striatal circuitry: from developmental aspects toward clinical applications. *Front. Cell. Neurosci.* 6:16. 10.3389/fncel.2012.00016 22529778 PMC3329876

[B136] Pencea V. Bingaman K. D. Freedman L. J. Luskin M. B. (2001a). Neurogenesis in the subventricular zone and rostral migratory stream of the neonatal and adult primate forebrain. *Exp. Neurol.* 172 1–16. 10.1006/exnr.2001.7768 11681836

[B137] Pencea V. Bingaman K. D. Wiegand S. J. Luskin M. B. (2001b). Infusion of brain-derived neurotrophic factor into the lateral ventricle of the adult rat leads to new neurons in the parenchyma of the striatum, septum, thalamus, and hypothalamus. *J. Neuro Ophthalmol.* 21 6706–6717. 10.1097/00041327-200206000-00020 11517260 PMC6763082

[B138] Pilz G. A. Carta S. Stäuble A. Ayaz A. Jessberger S. Helmchen F. (2016). Functional imaging of dentate granule cells in the adult mouse hippocampus. *J. Neurosci.* 36 7407–7414. 10.1523/jneurosci.3065-15.2016 27413151 PMC6705545

[B139] Podgorny O. Peunova N. Park J. H. Enikolopov G. (2018). Triple S-phase labeling of dividing stem cells. *Stem Cell Rep.* 10 615–626. 10.1016/j.stemcr.2017.12.020 29358087 PMC5830949

[B140] Pradillo J. M. Murray K. N. Coutts G. A. Moraga A. Oroz-Gonjar F. Boutin H. (2017). Reparative effects of interleukin-1 receptor antagonist in young and aged/co-morbid rodents after cerebral ischemia. *Brain Behav. Immun.* 61 117–126. 10.1016/j.bbi.2016.11.013 27856349 PMC5325120

[B141] Rakic P. Ayoub A. E. Breunig J. J. Dominguez M. H. (2009). Decision by division: making cortical maps. *Trends Neurosci.* 32 291–301. 10.1016/j.tins.2009.01.007 19380167 PMC3601545

[B142] Ramaswamy S. Goings G. E. Soderstrom K. E. Szele F. G. Kozlowski D. A. (2005). Cellular proliferation and migration following a controlled cortical impact in the mouse. *Brain Res.* 1053 38–53. 10.1016/j.brainres.2005.06.042 16051202

[B143] Reumers V. Deroose C. M. Krylyshkina O. Nuyts J. Geraerts M. Mortelmans L. (2008). Noninvasive and quantitative monitoring of adult neuronal stem cell migration in mouse brain using bioluminescence imaging. *Stem Cells* 26 2382–2390. 10.1634/stemcells.2007-2 18599812

[B144] Roe T. Reynolds T. C. Yu G. Brown P. O. (1993). Integration of murine leukemia virus DNA depends on mitosis. *EMBO J.* 12 2099–2108. 8491198 10.1002/j.1460-2075.1993.tb05858.x PMC413431

[B145] Saha B. Peron S. Murray K. Jaber M. Gaillard A. (2013). Cortical lesion stimulates adult subventricular zone neural progenitor cell proliferation and migration to the site of injury. *Stem Cell Res.* 11 965–977. 10.1016/j.scr.2013.06.006 23900166

[B146] Sakamoto M. Kageyama R. Imayoshi I. (2014). The functional significance of newly born neurons integrated into olfactory bulb circuits. *Front. Neurosci.* 8:121. 10.3389/fnins.2014.00121 24904263 PMC4033306

[B147] Satija R. Farrell J. A. Gennert D. Schier A. F. Regev A. (2015). Spatial reconstruction of single-cell gene expression data. *Nat. Biotechnol.* 33 495–502. 10.1038/nbt.3192 25867923 PMC4430369

[B148] Schmidt W. Reymann K. G. (2002). Proliferating cells differentiate into neurons in the hippocampal CA1 region of gerbils after global cerebral ischemia. *Neurosci. Lett.* 334 153–156. 12453618 10.1016/s0304-3940(02)01072-8

[B149] Shapiro E. M. Gonzalez-Perez O. Manuel García-Verdugo J. Alvarez-Buylla A. Koretsky A. P. (2006). Magnetic resonance imaging of the migration of neuronal precursors generated in the adult rodent brain. *Neuroimage* 32 1150–1157. 10.1016/j.neuroimage.2006.04.219 16814567 PMC4035244

[B150] Shapiro L. A. Ng K. Zhou Q. Y. Ribak C. E. (2009). SVZ-Derived newly generated neurons populate several olfactory and limbic forebrain regions. *Epilepsy Behav.* 14 74–80. 10.1016/j.yebeh.2008.09.011 18849007 PMC2677571

[B151] Shimada I. S. LeComte M. D. Granger J. C. Quinlan N. J. Spees J. L. (2012). Self-renewal and differentiation of reactive astrocyte-derived neural stem/progenitor cells isolated from the cortical peri- infarct area after stroke. *J. Neurosci.* 32 7926–7940. 10.1523/JNEUROSCI.4303-11.2012 22674268 PMC3398807

[B152] Shimada I. S. Peterson B. M. Spees J. L. (2010). Isolation of locally-derived stem/progenitor cells from the periinfarct area that do not migrate from the lateral ventricle after cortical stroke. *Stroke* 41 552–560. 10.1161/STROKEAHA.110.589010 20671247 PMC2937565

[B153] Snyder J. S. Cameron H. A. (2012). Could adult hippocampal neurogenesis be relevant for human behavior? *Behav. Brain Res.* 227 384–390. 10.1016/j.bbr.2011.06.024 21736900 PMC3210392

[B154] Song M. Yu S. P. Mohamad O. Cao W. Wei Z. Z. Gu X. (2017). Optogenetic stimulation of glutamatergic neuronal activity in the striatum enhances neurogenesis in the subventricular zone of normal and stroke mice. *Neurobiol. Dis.* 98 9–24. 10.1016/j.nbd.2016.11.005 27884724 PMC5315431

[B155] Sorrells S. F. Paredes M. F. Cebrian-Silla A. Sandoval K. Qi D. Kelley K. W. (2018). Human hippocampal neurogenesis drops sharply in children to undetectable levels in adults. *Nature* 555 377–381. 10.1038/nature25975.Human 29513649 PMC6179355

[B156] Spalding K. L. Bergmann O. Alkass K. Bernard S. Salehpour M. Huttner H. B. (2013). Dynamics of hippocampal neurogenesis in adult humans. *Cell* 153:1219. 10.1016/j.cell.2013.05.002 23746839 PMC4394608

[B157] Sumner J. P. Shapiro E. M. Maric D. Conroy R. Koretsky A. P. (2010). In vivo labeling of adult neural progenitors for mri with micron sized particles of iron oxide: quantitation of labeled cell phenotype. *Neuroimage* 44 671–678. 10.1016/j.neuroimage.2008.07.050 18722534 PMC2967480

[B158] Sun D. (2016). Endogenous neurogenic cell response in the mature mammalian brain following traumatic injury. *Exp. Neurol.* 275 405–410. 10.1016/j.expneurol.2015.04.017 25936874 PMC4627904

[B159] Sun W. Kim H. Moon Y. (2011). Control of neuronal migration through rostral migration stream in mice. *Anat. Cell Biol.* 43:269. 10.5115/acb.2010.43.4.269 21267400 PMC3026178

[B160] Sun X. Zhang Q. W. Xu M. Guo J. J. Shen S. W. Wang Y. Q. (2012). New striatal neurons form projections to substantia nigra in adult rat brain after stroke. *Neurobiol. Dis.* 45 601–609. 10.1016/j.nbd.2011.09.018 22005319

[B161] Sundholm-Peters N. L. Yang H. K. Goings G. E. Walker A. S. Szele F. G. (2005). Subventricular zone neuroblasts emigrat toward cortical lesions. *J. Neuropathol. Exp. Neurol.* 64 1089–1100. 16319719 10.1097/01.jnen.0000190066.13312.8f

[B162] Temple S. (2001). The development of neural stem cells. *Nature* 414 112–117. 10.1038/35102174 11689956

[B163] Tepper J. M. Bolam J. P. (2004). Functional diversity and specificity of neostriatal interneurons. *Curr. Opin. Neurobiol.* 14 685–692. 10.1016/j.conb.2004.10.003 15582369

[B164] Teramoto T. Qiu J. Plumier J. C. Moskowitz M. A. (2003). EGF amplifies the replacement of parvalbumin-expressing striatal interneurons after ischemia. *J. Clin. Invest.* 111 1125–1132. 10.1172/JCI200317170 12697732 PMC152938

[B165] Thored P. Arvidsson A. Cacci E. Ahlenius H. Kallur T. Darsalia V. (2006). Persistent production of neurons from adult brain stem cells during recovery after stroke. *Stem Cells* 24 739–747. 10.1634/stemcells.2005-0281 16210404

[B166] Tseng K. Y. Anttila J. E. Khodosevich K. Tuominen R. K. Lindahl M. Domanskyi A. (2018). MANF promotes differentiation and migration of neural progenitor cells with potential neural regenerative effects in stroke. *Mol. Ther.* 26 238–255. 10.1016/j.ymthe.2017.09.019 29050872 PMC5763030

[B167] Urbán N. Guillemot F. (2014). Neurogenesis in the embryonic and adult brain: same regulators, different roles. *Front. Cell. Neurosci.* 8:396 10.3389/fncel.2014.00396 PMC4245909 25505873

[B168] Vande Velde G. Raman Rangarajan J. Vreys R. Guglielmetti C. Dresselaers T. Verhoye M. (2012). Quantitative evaluation of MRI-based tracking of ferritin-labeled endogenous neural stem cell progeny in rodent brain. *NeuroImage* 62 367–380. 10.1016/j.neuroimage.2012.04.040 22677164

[B169] Vandeputte C. Reumers V. Aelvoet S. A. Thiry I. De Swaef S. Van den Haute C. (2014). Bioluminescence imaging of stroke-induced endogenous neural stem cell response. *Neurobiol. Dis.* 69 144–155. 10.1016/j.nbd.2014.05.014 24878507

[B170] Vreys R. Vande Velde G. Krylychkina O. Vellema M. Verhoye M. Timmermans J. P. (2010). MRI visualization of endogenous neural progenitor cell migration along the RMS in the adult mouse brain: validation of various MPIO labeling strategies. *NeuroImage* 49 2094–2103. 10.1016/j.neuroimage.2009.10.034 19850132

[B171] Wan F. Bai H. J. Liu J. Q. Tian M. Wang Y. X. Niu X. (2016). Proliferation and glia-directed differentiation of neural stem cells in the subventricular zone of the lateral ventricle and the migratory pathway to the lesions after cortical devascularization of adult rats. *BioMed. Res. Int.* 2016:3625959. 10.1155/2016/3625959 27294116 PMC4879261

[B172] Wang J. Fu X. Zhang D. Yu L. Lu Z. Gao Y. (2017). Effects of crenolanib, a nonselective inhibitor of PDGFR, in a mouse model of transient middle cerebral artery occlusion. *Neuroscience* 364 202–211. 10.1016/j.neuroscience.2017.09.025 28943249 PMC5653447

[B173] Wang Y. Jin K. Mao X. O. Xie L. Banwait S. Marti H. H. (2007). VEGF-overexpressing transgenic mice show enhanced post-ischemic neurogenesis and neuromigration. *J. Neurosci. Res.* 85 740–747. 10.1002/jnr 17243175

[B174] Winner B. Cooper-Kuhn C. M. Aigner R. Winkler J. Kuhn H. G. (2002). Long-term survival and cell death of newly generated neurons in the adult rat olfactory bulb. *Eur. J. Neurosci.* 16 1681–1689. 10.1046/j.1460-9568.2002.02238.x 12431220

[B175] Wojcik L. Sawicka A. Rivera S. Zalewska T. (2009). Neurogenesis in gerbil hippocampus following brain ischemia: focus on the involvement of metalloproteinases. *Acta Neurobiol. Exp.* 69 52–61. 19325641 10.55782/ane-2009-1729

[B176] Yamashita T. Ninomiya M. Hernández Acosta P. García-Verdugo J. M. Sunabori T. Sakaguchi M. (2006). Subventricular zone-derived neuroblasts migrate and differentiate into mature neurons in the post-stroke adult striatum. *J. Neurosci.* 26 6627–6636. 10.1523/JNEUROSCI.0149-06.2006 16775151 PMC6674034

[B177] Yan Y. P. Sailor K. A. Lang B. T. Park S. W. Vemuganti R. Dempsey R. J. (2007). Monocyte chemoattractant protein-1 plays a critical role in neuroblast migration after focal cerebral ischemia. *J. Cereb. Blood Flow Metab.* 27 1213–1224. 10.1038/sj.jcbfm.9600432 17191078

[B178] Yan Y. P. Lang B. T. Vemuganti R. Dempsey R. J. (2009a). Persistent migration of neuroblasts from the subventricular zone to the injured striatum mediated by osteopontin following intracerebral hemorrhage. *J. Neurochem.* 109 1624–1635. 10.1111/j.1471-4159.2009.06059.x 19457159

[B179] Yan Y. P. Sailor K. A. Lang B. T. Park S. W. Vemuganti R. Dempsey R. J. (2009b). Osteopontin is a mediator of the lateral migration of neuroblasts from the subventricular zone after focal cerebral ischemia. *Neurochem. Int.* 55 826–832. 10.1016/j.neuint.2009.08.007 19686792

[B180] Yang Z. Covey M. V. Bitel C. L. Ni L. Jonakait G. M. Levison S. W. (2007). Sustained neocortical neurogenesis after neonatal hypoxic/ischemic injury. *Ann. Neurol.* 61 199–208. 10.1002/ana.21068 17286251

[B181] Yang Z. Covey M. V. Bitel C. L. Ni L. Jonakait G. M. Levison S. W. (2008). Neonatal hypoxic/ischemic brain injury induces production of calretinin-expressing interneurons in the striatum. *J. Comp. Neurol.* 511 19–33. 10.1002/cne.21819 18720478 PMC3075928

[B182] Yang Z. Levison S. W. (2006). Hypoxia/ischemia expands the regenerative capacity of progenitors in the perinatal subventricular zone. *Neuroscience* 139 555–564. 10.1016/j.neuroscience.2005.12.059 16500031

[B183] Yi X. Jin G. Zhang X. Mao W. Li H. Qin J. (2013). Cortical endogenic neural regeneration of adult rat after traumatic brain injury. *PLoS One* 8:e070306. 10.1371/journal.pone.0070306 23922973 PMC3726380

[B184] Yoo S. W. Kim S. S. Lee S. Y. Lee H. S. Kim H. S. Lee Y. D. (2008). Mesenchymal stem cells promote proliferation of endogenous neural stem cells and survival of newborn cells in a rat stroke model. *Exp. Mol. Med.* 40 387–397. 10.3858/emm.2008.40.4.387 18779651 PMC2679267

[B185] Yoshikawa G. Momiyama T. Oya S. Takai K. Tanaka J. Higashiyama S. (2010). Induction of striatal neurogenesis and generation of region-specific functional mature neurons after ischemia by growth factors. *J. Neurosurg.* 113 835–850. 10.3171/2010.2.JNS09989 20345217

[B186] Zhang C. Chopp M. Cui Y. Wang L. Zhang R. Zhang L. (2010). Cerebrolysin enhances neurogenesis in the ischemic brain and improves functional outcome after stroke. *J. Neurosci. Res.* 88 3275–3281. 10.1002/jnr.22495 20857512 PMC3073377

[B187] Zhang F. Duan X. Lu L. Zhang X. Zhong X. Mao J. (2016). In vivo targeted MR imaging of endogenous neural stem cells in ischemic stroke. *Molecules* 21:E1143. 10.3390/molecules21091143 27589699 PMC6273863

[B188] Zhang P. B. Liu Y. Li J. Kang Q. Y. Tian Y. F. Chen X. L. (2005). Ependymal/subventricular zone cells migrate tothe peri-infarct region and differentiate into neurons and astrocytes after focal cerebral ishemia in adult rats. *J. First Mil. Med. Univ.* 25 1201–1206. 16234089

[B189] Zhang R. Xue Y. Y. Lu S. D. Wang Y. Zhang L. M. Huang Y. L. (2006). Bcl-2 enhances neurogenesis and inhibits apoptosis of newborn neurons in adult rat brain following a transient middle cerebral artery occlusion. *Neurobiol. Dis.* 24 345–356. 10.1016/j.nbd.2006.07.012 16996745

[B190] Zhang R. Zhang Z. Wang L. Wang Y. Gousev A. Zhang L. (2004). Activated neural stem cells contribute to stroke-induced neurogenesis and neuroblast migration toward the infarct boundary in adult rats. *J. Cereb. Blood Flow Metab.* 24 441–448. 10.1097/00004647-200404000-00009 15087713

[B191] Zhang R. L. Chopp M. Gregg S. R. Toh Y. Roberts C. Letourneau Y. (2009). Patterns and dynamics of subventricular zone neuroblast migration in the ischemic striatum of the adult mouse. *J. Cereb. Blood Flow Metab.* 29 1240–1250. 10.1038/jcbfm.2009.55 19436318 PMC2741163

[B192] Zhang R. L. Chopp M. Roberts C. Jia L. Wei M. Lu M. (2011). Ascl1 lineage cells contribute to ischemia-induced neurogenesis and oligodendrogenesis. *J. Cereb. Blood Flow Metab.* 31 614–625. 10.1038/jcbfm.2010.134 20736965 PMC3049516

[B193] Zhang R. L. LeTourneau Y. Gregg S. R. Wang Y. Toh Y. Robin A. M. (2007). Neuroblast division during migration toward the ischemic striatum: a study of dynamic migratory and proliferative characteristics of neuroblasts from the subventricular zone. *J. Neurosci.* 27 3157–3162. 10.1523/JNEUROSCI.4969-06.2007 17376977 PMC6672487

[B194] Zhao M. Momma S. Delfani K. Carlen M. Cassidy R. M. Johansson C. B. (2003). Evidence for neurogenesis in the adult mammalian substantia nigra. *Proc. Natl. Acad. Sci. U.S.A.* 100 7925–7930. 10.1073/pnas.1131955100 12792021 PMC164689

[B195] Zhong X. M. Zhang F. Yang M. Wen X. H. Zhang X. Duan X. H. (2015). In vivo targeted magnetic resonance imaging of endogenous neural stem cells in the adult rodent brain. *BioMed. Res. Int.* 2015:131054. 10.1155/2015/131054 26583085 PMC4637027

